# A previously unknown cyclic alkanolamine and molecular ranking using the pair distribution function

**DOI:** 10.1107/S2052520621010088

**Published:** 2021-11-17

**Authors:** Gianpiero Gallo, Maxwell W. Terban, Igor Moudrakovski, Tatjana Huber, Martin Etter, Martin Ernst, Bernd Hinrichsen, Robert E. Dinnebier

**Affiliations:** a Max Planck Institute for Solid State Research, Heisenbergstraße 1, 70569 Stuttgart, Germany; b Department of Chemistry and Biology "A. Zambelli", University of Salerno, Via Giovanni Paolo II, 132, 84084 Fisciano (SA), Italy; c BASF SE, Carl-Bosch-Strasse 38, 67056 Ludwigshafen am Rhein, Germany; d Deutsches Elektronen-Synchrotron (DESY), Notkestrasse 85, 22607 Hamburg, Germany

**Keywords:** heterocycle, X-ray powder diffraction, pair distribution function, structure solution, co-refinement

## Abstract

A new six-membered cyclic alkanolamine with chemical formula C_6_H_15_N_3_O_3_ was synthesized by the reaction of glycolaldehyde with gaseous ammonia. The molecular structure, characterized by a hexagonal ring of alternating carbon and nitro­gen atoms with three hy­droxy­methyl groups attached to the carbon atoms, could not be unambiguously determined by elemental analysis and ^1^H/^13^C/^15^N NMR. The molecular structure and conformation were further determined using a combination of vibrational spectroscopy (IR and Raman) and real-space pair distribution function (PDF) analysis. The crystal structure was determined *ab initio* from laboratory X-ray powder diffraction (XRPD) with orthorhombic space group *Ama*2 (No. 40) and unit-cell parameters *a* = 12.1054 (2) Å, *b* = 13.5537 (2) Å and *c* = 5.20741 (8) Å. Consistent structure models could be obtained by symmetry-independent PDF and PDF-Rietveld co-refinements. Independent local structure refinements indicate that the most likely deviations from the average structure consist of small tilting and translational distortions of hydrogen-bonded molecular stacks. Thermal analysis (TG/DTA) and temperature-dependent XRPD measurements were also performed to determine the thermal behavior.

## Abstract 2

The crystal structure of a previously unknown small heterocyclic alkanolamine was determined by X-ray powder diffraction. Total scattering analysis of small organic molecules is shown to be useful to help disambiguate spectroscopic and elemental analyses.

## 1. Introduction

Amines are an extensive and important class of organic compounds. They are formally derivatives of ammonia since they can be obtained by replacing one or more hydrogen atoms with different organic substituents (McMurry, 1992 ▸). Due to their chemical properties, amines are versatile compounds with applications as building blocks for amino acids, pharmaceutical drugs, herbicides, agrochemicals, polymer additives and surfactants, and are, therefore, of considerable industrial importance (Roose *et al.*, 2015 ▸).

Starting over a century ago, an increasing number of synthetic routes for the production of new amino compounds have been designed using small precursors like ammonia and aldehydes. As an example, the combination of ammonia and formaldehyde (Butlerow, 1860 ▸; Baur & Rüetschi, 1941 ▸), the smallest aldehyde, results in the formation of hexamine, which is used in the production of resins, fuel tablets, explosives, vulcanization accelerators, anticorrosion agents, antibacterial agents and preservatives (Roose *et al.*, 2015 ▸). When aliphatic aldehydes are used in the presence of ammonia, linear or cyclic amino compounds can be formed, such as alkanolamine or hexa­hydro­triazine (Roose *et al.*, 2015 ▸). An example of cyclic amine formation is the reaction of acetaldehyde with ammonia yielding crystalline tri­ethyl­hexa­hydro­triazine trihydrate (Aschan, 1915 ▸; Lund, 1951 ▸).

Most of these compounds are obtained as solids, and their molecular structures were determined by NMR spectroscopy (Nielsen *et al.*, 1979 ▸; Xue *et al.*, 2011 ▸; Sun *et al.*, 2011 ▸; Ghandi *et al.*, 2006 ▸) or by structure solution using single-crystal X-ray diffraction (SC-XRD) (Lund, 1951 ▸; George & Gilardi, 1987 ▸; Giumanini *et al.*, 1985 ▸; Mallo *et al.*, 2018 ▸). The latter technique generally requires recrystallization from solution, though this is not always possible (Nielsen *et al.*, 1974 ▸). The crystal structure can also be determined by X-ray powder diffraction (XRPD) (Pagola *et al.*, 2001 ▸; Chan *et al.*, 1999 ▸), but this method requires prior knowledge of the molecular structure.

The pair distribution function (PDF), obtained from total scattering measurement, is another way to access information about both the short- and long-range structuring within a material. It directly gives the distribution of atom-pair distances and the extent of molecular ordering, independent of any assumptions about crystallographic symmetry, even for amorphous materials (Billinge *et al.*, 2010 ▸; Terban *et al.*, 2015 ▸). For special cases, PDF has been shown to contain sufficient information for *ab initio* molecular structure solution (Juhás *et al.*, 2006 ▸), though for most organic molecules, only the first two or three nearest neighbor distances can be distinctly resolved. In this case, it can still be used to test the plausibility of a particular molecular structure and conformation (Benmore *et al.*, 2013 ▸). It has been less frequently applied to investigate the presence or type of local deviations from the average structure for organic crystals (Pütz *et al.*, 2020 ▸; Schlesinger *et al.*, 2020 ▸).

Herein, we present the determination of a new cyclic amino alcohol compound, glycolaldehyde ammonia [IUPAC: (1,3,5-triazine-2,4,6-triyl)tri­methanol (1)], which was recently synthesized at BASF SE by reaction of glycolaldehyde and ammonia. We provide a comprehensive investigation of this novel, small organic molecule including elemental analysis (EA), liquid and solid-state ^1^H/^13^C/^15^N NMR, infrared (IR) and Raman spectroscopy, XRPD structure solution, PDF analysis and SEM. The thermal behavior was also investigated by thermogravimetric analysis (TGA), differential thermal analysis (DTA) and temperature-dependent XRPD measurements. In particular, we show that PDF analysis can be especially useful for resolving ambiguities of elemental analysis and spectroscopies in molecular structure assignment for small molecules and give additional information about likely molecular conformations. We also discuss the implications of real- and reciprocal-space co-refinement for obtaining more robust structure solutions of small molecules.

## 2. Experimental

### 2.1. Synthesis process

The synthesis of (1) was performed at BASF SE. Gaseous glycolaldehyde was provided by evaporation from an aqueous solution of the glycolaldehyde dimer in THF (7.5 wt% glycolaldehyde dimer, 11.5 wt% THF, 80 wt% water and 1 wt% tetraglyme) by heating the solution to 160°C in a tube evaporator comprised of Raschigrings (flow rate: 70.7 g h^−1^). The gas was fed into an unheated reaction chamber operated at ambient pressure. Gaseous ammonia (20 normal litres per hour) at room temperature was also fed to the reaction chamber through a separate inlet. Colorless crystals desublimated at the cooler parts of the reaction chamber, and a yellow-brownish clear solution condensed from the gas phase at the bottom. Over two hours, 138.1 g of raw product was obtained. The total yield of conversion products of glycolaldehyde and ammonia, including the triazinane, was about 73% (as determined by GC analysis). ^1^H NMR (400 MHz, D_2_O) δ4.69 (*s*, broad, 3H), δ3.69 (*t*, *J* = 4.0 Hz, 3H), δ3.465 (*d*, *J* = 4.0 Hz, 6H), δ2.2 (*s*, broad, 3H); ^1^H NMR (400 MHz, DMSO-d_6_) δ4.72 (*t*, 5.2, 3H), δ3.56 (*t*, *J* = 3.6 Hz, 3H), δ3.37 (*dd*, *J* = 3.9 Hz, 6H), δ1.61 (*s*, broad, 3H); ^13^C{^1^H} NMR (100.5 MHz, D_2_O) δ68.8 (*d*, *J* = 146.1 Hz), δ63.4 (*t*, *J* = 143.1 Hz); ^15^N{^1^H} NMR (40 MHz, D_2_O) δ-320.2. Patent WO/2020/249428 awarded to Ernst *et al.* (2020 ▸).

### 2.2. Elemental analysis

The amount of oxygen was determined using a EURO EA 3000 (EuroVector/HekaTech) analyser. Carbon, hydrogen and nitrogen content was determined using a Vario MICRO CUBE (Elementar). From the combined results, the following elemental composition was obtained: C 40.67, H 8.53, N 23.71 and O 27.09 wt%.

### 2.3. NMR spectroscopy

NMR spectra of solutions of (1) in D_2_O (^1^H, ^13^C and ^15^N NMR) and DMSO-d_6_ (^1^H NMR) have been obtained on a Jeol ECS400s spectrometer in a magnetic field of 9.4 T at resonance frequencies of 400.13, 100.53 and 40.56 MHz. Field stabilization was accomplished on the signals of deuterated solvents (D_2_O and DMSO-d_6_). Signals of tetramethylsilane (TMS) and nitromethane (CH_3_NO_2_) were used for the referencing in ^1^H, ^13^C and ^15^N spectra (in all cases δ_iso_ = 0.0 ppm). ^13^C and ^15^N solid state NMR measurements were performed on a Bruker Avance-III 400 MHz instrument at magnetic field of 9.4 T at the same resonance frequencies as above. Standard Bruker BL2.5 (rotors of 2.5 mm OD) and BL4 (rotors of 4.0 mm OD) double resonance Magic Angle Spinning (MAS) probes were used, with the samples packed in ZrO_2_ ceramic spinners and spun at 30 kHz (^1^H), 12 kHz (^13^C), and 5 kHz (^15^N). ^1^H MAS spectrum was obtained with simple Bloch-decay pulse sequence. ^13^C and ^15^N MAS spectra were obtained using cross polarization (CP) from proton. The CP experiment was implemented with a ramped contact pulse on proton channel and accompanied by high power proton decoupling. The cross-polarization conditions have been tuned on signals from solid α-glycine (^13^C, ^15^N), which have also served as secondary chemical shift standards (^15^N δ_iso_ = −347.58 ppm, ^13^C δ_iso_ = 176.46 ppm) (Bryce *et al.*, 2001 ▸). The experiments have been optimized both for cross-polarization contact times (2 ms for ^13^C and 6 ms for ^15^N) and repetition delays (120 s for ^1^H and 40 s for ^13^C and ^15^N). A total of 16 (^1^H) to 4000 accumulations (^13^C and ^15^N) were collected for a sufficiently good signal-to-noise ratio. Assignments in the spectra were aided by ACD/NMR predictor (liquids) (ACD, 2019 ▸) and DFT calculations of the chemical shielding using CASTEP quantum mechanical computational package (Clark *et al.*, 2005 ▸; Yates *et al.*, 2007 ▸) as implemented in BIOVIA *Materials Studio* (Dassault Systèmes, 2018 ▸), using the experimental crystal structure.

### 2.4. Infrared spectroscopy (IR)

The infrared spectrum of (1) was recorded in attenuated total reflection geometry on a PerkinElmer Spectrum Two FT-IR spectrometer equipped with a diamond crystal. The background spectrum was measured separately and subtracted. Tentative band assignment for (1) can be found in Table 1 ▸

### 2.5. Raman spectroscopy

The Raman spectrum of (1) was recorded in transmission geometry using a Horiba iHR320 imaging spectrometer with a laser (λ = 633 nm) as excitation source. Tentative band assignment for (1) can be found in Table 1 ▸.

### 2.6. Scanning electron microscopy

Scanning electron microscope (SEM) images of (1) were obtained with a TESCAN Vega TS 5130 MM instrument equipped with X-MaxN 20 SDD attachment (Oxford Instruments). The sample was sputtered with gold nanoparticles before the analysis.

### 2.7. Thermal analysis

Thermal analyses were carried out using an STA 449 F5-Jupiter (Netzsch) device for TG and DTA measurements. The sample was placed in an Al_2_O_3_ crucible and heated up from 30°C to 800°C at a heating rate of 5°C min^−1^ in a 20 ml min^−1^ O_2_ stream. An empty crucible was used as reference material. The TG curve was corrected using the data of a standard (Al_2_O_3_), which was measured with the same temperature program.

### 2.8. Laboratory X-ray powder diffraction (XRPD)

The XRPD pattern of (1) used for crystal structure solution and Rietveld refinement was collected at room temperature on a laboratory powder diffractometer in Debye–Scherrer geometry [Stadi-P Diffractometer (Stoe), Cu-*K*α1 radiation from primary Ge(111)-Johann-type monochromator, three Mythen 1K detectors (Dectris)]. The sample was gently ground and filled into a 0.5 mm diameter borosilicate glass capillary (WJM-Glas/Mueller GmbH), which was spun during the measurement. A total scan time of 12 h was applied and the pattern was measured over a 2θ range from 0° to 110°.

Temperature-dependent XRPD measurements were carried out on a Bruker D8-Advance powder diffractometer in Debye–Scherrer geometry with Cu-*K*α1 radiation from a primary Ge(111)-Johannson-type monochromator and Våntec detector. The sample was loaded into a 0.7 mm diameter glass capillary, which was spun during the measurements. The patterns were measured over a 2θ range from 2.0° to 60.0°. A total scan time of 3 h was applied per measurement. The temperature was adjusted using a TC transmission furnace (mri). The sample was heated from 30°C to 150°C in 20°C steps with a heating rate of 5°C min^−1^. During each step, a diffraction pattern was collected, after a delay time of 5 min to ensure thermal equilibration of the sample.

### 2.9. Structure determination and Rietveld refinement

Crystal structure solution and Rietveld refinement (Rietveld, 1969 ▸) of the crystal structure were performed with the program *TOPAS 6.0* (Coelho, 2017 ▸). Indexing was carried out by an iterative use of singular value decomposition (LSI) as implemented in *TOPAS 6.0* (Coelho, 2003 ▸). The crystal structure was determined by applying the global optimization method of simulated annealing (SA) (Coelho, 2000 ▸) in real space.

The peak profile was determined by a Pawley refinement (Pawley, 1981 ▸) using the fundamental parameter approach as implemented in *TOPAS* (Cheary *et al.*, 2004 ▸). The background was modeled using Chebyshev polynomials. The small hump in the background of the diffraction pattern caused by the glass capillary was modeled with a very broad Lorentzian-type peak. The molecule was described using rigid bodies in *z*-matrix notation and their rotation and translation modes were refined. The bond lengths and angles were taken from a related crystal structure (Lund, 1951 ▸). All hydrogen atoms, except for the hydroxyl hydrogens which were not included, were fixed at geometric calculated positions and an overall isotropic displacement parameter was applied for all atoms. As suggested by the indexing process, the structure was solved in *C*2 space group with the same unit-cell parameters as in Table 2 ▸ and β angle equal to 90°. The right space group was determined after a visual inspection of the crystal structure and the use of the software *PLATON* (Spek, 2009 ▸), which gives the higher symmetry space group *Ama*2. Eventually, the background, unit-cell parameters, rotations, translations, and bond lengths and angles of the rigid body were refined without any constraints during the final Rietveld refinement. The final agreement factors are listed in Table 2 ▸. The atomic coordinates and selected bond distances are given in Tables S3 and S4.

### 2.10. Pair distribution function

Experiments were carried out using beamline P02.1 at PETRA III (DESY). The diffraction dataset was collected at room temperature using a 2D Perkin Elmer XRD1621 (2048 × 2048 pixels and 200 µm × 200 µm pixel size) with sample-to-detector distance of 303.660 mm (Chupas *et al.*, 2003 ▸). The incident wavelength of the X-rays was λ = 0.207 Å (60.0 keV). Calibration of the experimental setup was performed using a silicon standard sample. The investigated powder was loaded into a 1 mm Kapton capillary tube. The background was determined using an empty Kapton capillary tube.

Raw 2D data were corrected for geometrical effects and polarization, then azimuthally integrated to produce 1D scattering intensities versus the magnitude of the momentum transfer *Q* (where *Q* = 4πsinθ/λ for elastic scattering) using the program *Fit2D* (Hammersley *et al.*, 1996 ▸). The program *xPDFsuite* (Juhás *et al.*, 2013 ▸; Yang *et al.*, 2014) ▸ was used to perform the background subtraction, further corrections, and normalization to obtain the reduced total scattering structure function, *F*(*Q*), and Fourier transformation to obtain the pair distribution function (PDF), *G*(*r*).

## 3. Results and discussion

### 3.1. Investigation of the molecular structure

**Figure 1:**
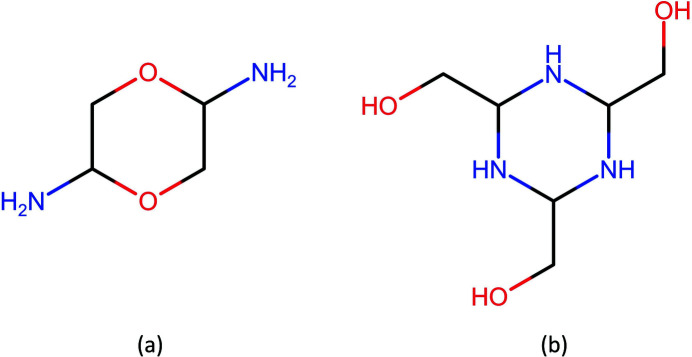
Possible molecular structures of (1).

The molecular structure of (1) was initially assessed by EA and solution ^1^H/^13^C/^15^N NMR in D_2_O due to the high solubility of (1) in this solvent. Although the exact molecular composition could not be unambiguously ascertained, the general chemical formula C_2*n*_H_5*n*_O_*n*_N_*n*_·*x*H_2_O was determined based on EA. The identification of structural segments by NMR, such as *XY*-CH-CH_2_-*Y* with *X* and *Y* heteroatoms (Figs. S1–S4), led to two possible candidates shown in Fig. 1 ▸. Both have six-membered rings incorporating heteroatoms.

The first candidate (with composition C_4_H_10_N_2_O_2_), which was previously proposed (Kitagawa *et al.*, 1987 ▸), has two oxygen atoms in the ring with two primary amino groups attached to the carbon atoms. The second candidate (with composition C_6_H_15_N_3_O_3_) has three secondary amines condensed in the ring with three hydroxymethyl groups attached to the carbon atoms. A broad, poorly resolved peak of residual protons of D_2_O at around 4.69 ppm in the ^1^H NMR spectrum obscures signals from hydroxyl protons (Fig. S1) and does not allow for an unambiguous differentiation between the models. ^1^H NMR spectrum of (1) in DMSO-d_6_, despite the low solubility, shows improved resolution both for hydroxyl and amino groups (Fig. S4). The character of *J*-coupling of the signal at 4.72 ppm points to connection of the hydroxyls to the methylene groups. Still somewhat broadened, but well resolved, the signal at 1.61 ppm belongs to N—H protons, further supporting the second model. The most likely reason for a severe broadening of the N—H signal in the spectrum recorded in D_2_O is due to the hydrogen bonding between amino group and water. In addition, we note here that XRPD and thermal analysis showed that there is no structural water present, as detailed later.

**Figure 2:**
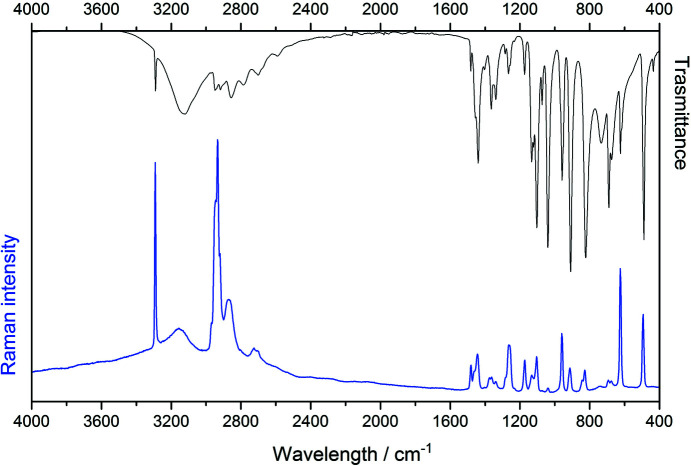
IR (black) and Raman (blue) spectra of (1).

The IR and Raman spectra of (1), shown in Fig. 2 ▸, were collected in order to give further details about the molecular structure and to support or exclude the proposed models. A tentative band assignment (Table 1 ▸) was performed using spectroscopic data of related structures (Neelakantan, 1963 ▸; Hirokawa *et al.*, 1980 ▸; Kobayashi *et al.*, 1976 ▸; Dwarakanath & Sathyanarayana, 1979 ▸; Isac Paulraj & Muthu, 2013 ▸; Thomas *et al.*, 2005 ▸; Nielsen *et al.*, 1973 ▸; Tuguldurova *et al.*, 2017 ▸). Although we were not able to assign all the bands, several features related to one of the proposed structures can be confirmed. In the high wavenumber region of the spectra, the band at 3290 cm^−1^ can be attributed to the presence of OH and NH stretching modes. Broad bands with maxima at 3129 and 3153 cm^−1^, in IR and Raman spectra respectively, can be assigned to NH or OH-stretching modes. The absence of bands in the region between 2400–1600 cm^−1^ excludes the presence of an aromatic ring (C—H bending, C=C stretching), carbonyl group (C=O stretching) and primary amine (N—H_2_ bending). This information, along with the presence of CH_2_, COH, and HCCN bending modes attributable to the hydroxymethyl group, also supports the second model.

**Figure 3:**
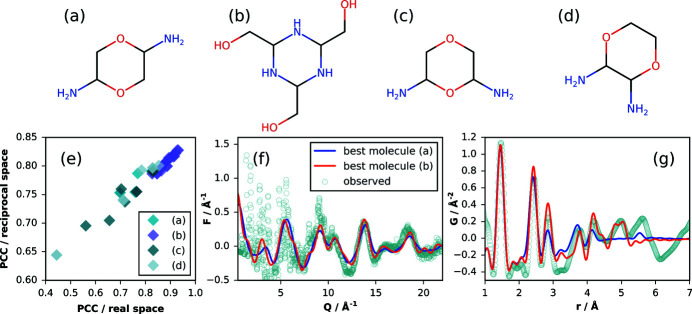
The four molecular structures investigated by conformation analysis (*a*)–(*d*) and ranked by Pearson correlation coefficient (PCC) comparison to the experimental *F*(*Q*) and *G*(*r*) functions (*e*). Experimental and simulated patterns from the top performing conformations of molecules (*a*) and (*b*) are compared for *F*(*Q*) and *G*(*r*) (*f*, *g*) respectively.

The molecular structure was further investigated by total scattering and PDF analysis. Starting with four different molecular structures satisfying the stoichiometric composition [Figs. 3 ▸(*a*)–3 ▸(*d*)], including those ruled out by spectroscopy, a set of conformers was generated using *Mercury* from the Cambridge Structural Database (CSD) (Macrae *et al.*, 2020 ▸). The resulting molecules, 50 in total, were further relaxed using the MMFF94s force field (Halgren, 1999 ▸). Then, the reduced total scattering structure function *F*(*Q*) and the PDF *G*(*r*) were simulated for every molecule using the Debye scattering equation calculator in *Diffpy-CMI* (Juhás *et al.*, 2015 ▸) and ranked against the experimental patterns by calculation of the Pearson correlation coefficient (PCC) (Billinge *et al.*, 2010 ▸; Terban *et al.*, 2015 ▸).

The range of comparison used for *F*(*Q*) was *Q* = 6.0–22.0 Å^−1^ to minimize the amount of overlap with substantial intermolecular signal (Mou *et al.*, 2015 ▸). For *G*(*r*), a range of *r* = 1.9–5.25 Å was used. We found that the sensitivity of the PCC values to the structural differences was better for PDF comparison, as shown in Fig. 3 ▸(*e*), and could be further improved by not including the nearest neighbor peak, which is nearly identical for these four molecules. The maximum of 5.25 Å was chosen as the distance beyond which intramolecular contributions to the PDF were minimal for all molecules. In general, the conformers of the molecule with composition C_6_H_15_N_3_O_3_ performed overwhelmingly better than the other candidates by both reciprocal- and real-space comparison, in agreement with the previous results. In addition, many structures could be ruled out by visual inspection of disagreement in the third nearest neighbor distance distribution. Figs. 3 ▸(*f*) and 3 ▸(*g*) show the comparisons of the experimental patterns to those simulated for the top performing conformers for the two different molecular candidates shown in Fig. 1 ▸. The rankings suggest that the best candidate consists of a cyclic chair conformation with the hydroxymethyl groups all pointing up perpendicular to the ring, though a few candidates with one hydroxymethyl group twisted to the side, near the plane of the ring, performed almost as well. The effects of relative position of the hydroxymethyl groups in the top candidate with composition C_6_H_15_N_3_O_3_ are demonstrated in Fig. S6.

### 3.2. Structure determination

**Figure 4:**
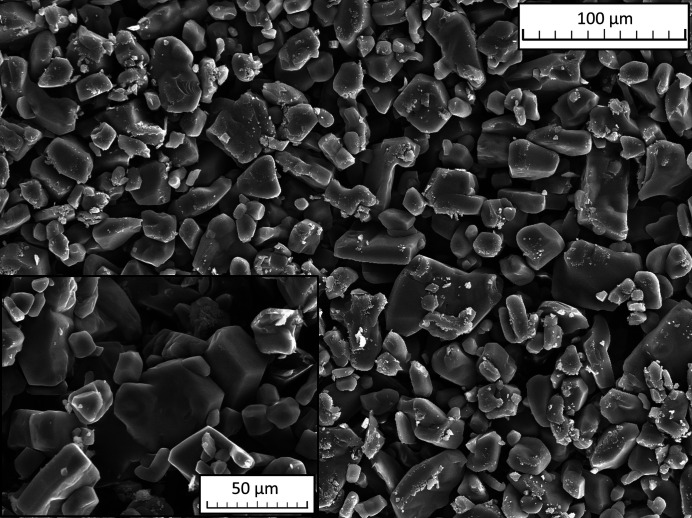
SEM image of crystallites of (1) with dimensions between 5 and 60 µm.

**Figure 5:**
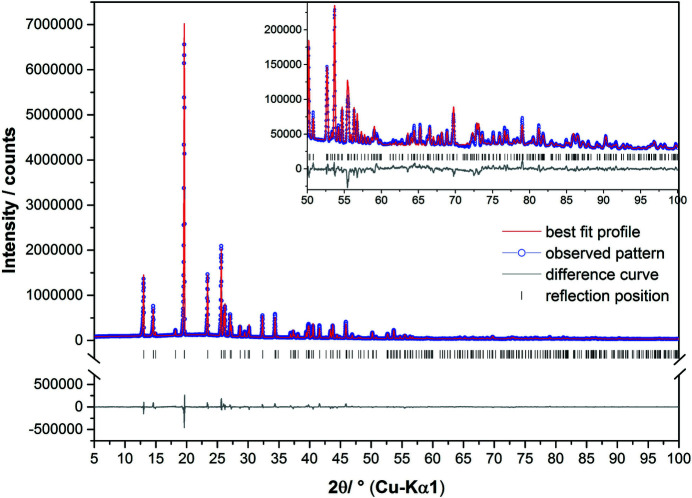
Diffraction pattern of (1) at ambient conditions. The observed pattern (circles), the best Rietveld fit profile (line) and the difference curve between the observed and the calculated profiles (below) are shown. The high angle part starting at 50° in 2θ is enlarged for clarity.

**Figure 6:**
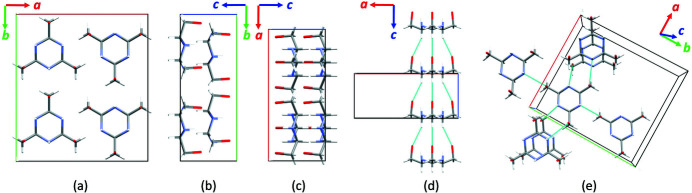
Projections of the crystal structure of (1) along the crystallographic *a*-, *b*- and *c*-axis (*a*)–(*c*), and representation of the intracolumnar hydrogen bonds along *b*-axis (*d*) and inter-columnar hydrogen bond along *c*-axis (*e*).

Since SEM images showed the presence of faceted crystallites with dimensions ranging from 5 to 60 µm in the bulk material (Fig. 4 ▸), XRPD analysis was performed on (1) in order to determine the crystal structure and further confirm the molecular model. Structure solution from XRPD confirmed the molecular structure and conformation and revealed that (1) crystallizes in the polar orthorhombic space group *Ama*2 [*a* = 12.1054 (2) Å, *b* = 13.5537 (2) Å, *c* = 5.20741 (8) Å, *V* = 854.40 (2) Å^3^, Table 2 ▸]. The fit of the whole powder pattern is shown in Fig. 5 ▸. Figs. 6 ▸(*a*)–6 ▸(*c*) shows the projections of the resulting structure along the crystallographic *a*, *b* and *c* axes. The structure contains four total molecules (*Z* = 4) and one independent molecule that sits on a mirror plane, so only half of the molecule is required in the asymmetric unit (*Z*′ = 0.5). This was defined as a rigid body with six atoms on the mirror plane and nine atoms on general positions. The unit cell contains four molecules with the hexagonal backbone ring parallel to the *ab* plane and the hydroxymethyl group almost parallel to the *c*-axis. Mirror planes perpendicular to the *a*-axis run through the center of the rings. The molecules are stacked along the crystallographic *c*-axis forming a set of parallel columns with a separation distance equal to *c*. Due to the close packing of the molecules, the crystal structure does not show any voids (probe radius: 1 Å) likely to contain structural water.

A dense hydrogen-bonding network is present in the crystal structure. C—H⋯O hydrogen bonds (C⋯O distances: 3.51 Å, C—H⋯O angles: 152.8–152.6°) involving the hydrogen attached to the carbon atoms in the backbone ring and the hydroxyl group related oxygen atom are responsible for the intra-column interactions [Fig. 6 ▸(*d*)]. Intercolumnar interactions are made up of N—O hydrogen bonds (distance: 2.8–2.9 Å) involving the nitrogen atom of the backbone ring and the hydroxyl oxygen atom [Fig. 6 ▸(*e*)]. These interactions allow each molecule to interact with six surrounding molecules.

^13^C and ^15^N CP MAS solid state NMR (Fig. S5) support the crystal structure model obtained by powder diffraction. In particular, the observed 2:1 intensity ratio of the two peaks in ^15^N NMR corresponds to the occupancies of the two crystallographically independent nitrogen atoms in the structure.

### 3.3. Local structure refinement

The combination of high symmetry space group and the use of a rigid body as scaffold for the molecule in the structure solution process imposes strict limitations on both bond and torsion angles, and intermolecular interactions. Therefore, to verify the local structure and reveal any deviation from the average crystal structure obtained from XRPD analysis, several refinements to the PDF curve were carried out, systematically lowering the space group symmetry from orthorhombic (*Ama*2), to monoclinic (*C*2), and to triclinic (*P*1), using the program *TOPAS 6.0* (Coelho *et al.*, 2015 ▸). The first two groups are related by group–subgroup relationship where *Ama*2 can be transformed to *C*2 by losing all mirror and glide planes (a change in the order of the unit-cell parameters was applied to avoid the nonstandard space group *A*2). *P*1 represents the space group without any symmetry except the identity. The number of molecules in the asymmetric unit goes from *Z*′ = 0.5 to 1.0 to 4.0, allowing for changes in relative positioning of the neighboring molecules. For *C*2 and *P*1, the hydroxymethyl torsion angles can be refined in the absence of the mirror plane.

In all three cases, bond lengths, and bond and torsion angles were allowed to refine by type when not constrained by symmetry. Strategies for modeling the correlated motion of covalently bonded versus less strongly interacting intermolecular atom-pairs have been described (Rademacher *et al.*, 2012 ▸; Prill *et al.*, 2015 ▸; Terban *et al.*, 2020 ▸). Here, we used two different isotropic displacement factors for the non-hydrogen (*B*_eq1_) and hydrogen atoms (*B*_eq2_) to describe the intermolecular pair peak-widths. One additional parameter (*B*_intra1_) was employed to describe the peak-widths for intramolecular pairs containing non-H atoms and *X*—H bonds. The PDF refinements were performed over both short (1.2–15.0 Å) and long (1.2–70.0 Å) ranges, and the refined parameters and fits are given in Table S1 and Figs. S7–S12, respectively.

**Figure 7:**
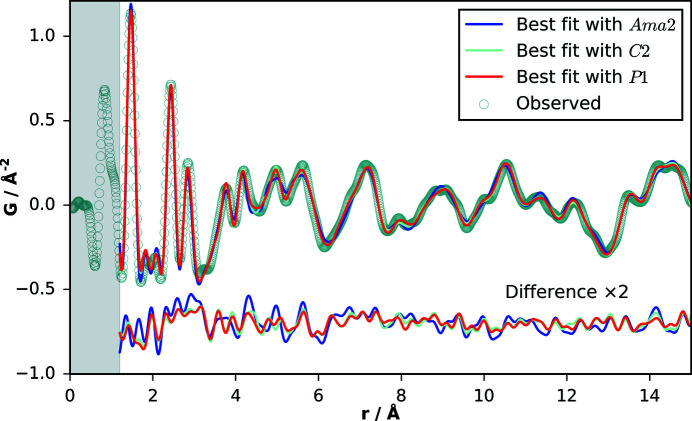
PDF fits of the models with space groups *Ama*2, *C*2 and *P*1 over a range of *r* = 1.2–15 Å. Similar improvement is observed for the *C*2 and *P*1 models. The shaded area represents the region dominated by systematic effects.

On refinement of the *Ama*2 model, inspection of the shorter-*r* part of the PDF confirms the contributions to intra- and intermolecular features as suggested by the molecular conformation analysis. The peak at *ca* 1.46 Å corresponds to C—C/C—N/C—O bonds; the peaks at around 2.43 Å and 2.85 Å correspond to atomic pairs in the backbone ring and C—N_ring_/O—C_ring_/O—N_ring_ pairs, and peaks at 3.78, 4.19, and 4.99 Å correspond to C—C_ring_, O—C_ring_ and O—C pairs, respectively. We excluded the features around 0.83–1.05 Å from the analysis: these are primarily artifacts resulting from systematic errors due to truncation effects in the Fourier transformation and slowly varying corrections applied in the data normalization (Juhás *et al.*, 2013 ▸; Peterson *et al.*, 2003 ▸). The shoulder at 1.05 Å may contain information about C/N/O—H bond pairs (expected average distances: C—H ∼ 1.09 Å, N—H ∼ 1.0 Å and O—H ∼ 0.9 Å (Fox & Whitesell, 1995 ▸), though this was unclear and not further investigated here. While the fit for *Ama*2 was quite good for typical organic materials (*R*_wp_ = 14.6%), it produced slightly broader than expected features at 5.0 and 5.5 Å where intermolecular contributions become much more significant. These features could be fitted with significant and similar improvement using *C*2 and *P*1 (*R*_wp_ = 10.3/9.8%, Fig. 7 ▸). The refined molecular conformations were consistent across all refinements, (Figs. S13–S15) whereas an appreciable change in the intermolecular arrangement of neighboring molecules in the unit cell suggests that slight, local distortions from the on-average crystallographic orientations exist in the form of small tilts and translations of segments of the hydrogen-bonded stacks (Fig. S16). The three models, obtained from PDF refinements, were then also re-refined against the XRPD data (Table S2 and Figs. S17–S19). Compared to the XRPD refinement from structure determination, the fit quality for the three models did not substantially improve, which in addition to the absence of additional Bragg peaks for the lower symmetries, suggests that the observed distortions are only short-range ordered.

XRPD/PDF co-refinements were performed with the aim of finding the global optimum based on precise uni-cell parameter information and average molecular arrangement from reciprocal space, and local molecular arrangements, conformation, and bond distances in real space. The refined crystallographic parameters for the symmetry-independent model are listed in Table 3 ▸, and choice of data weighting factors are shown in Fig. S20. The results show that PDF data provide complementary and substantial information for improving the robustness of XRPD structure solution, even in the absence of symmetry assumptions, as has also been indicated by recent progress toward structure solution of molecular crystal structures from the PDF (Prill *et al.*, 2016 ▸; Schlesinger *et al.*, 2021 ▸).

### 3.4. Thermal behavior

**Figure 8:**
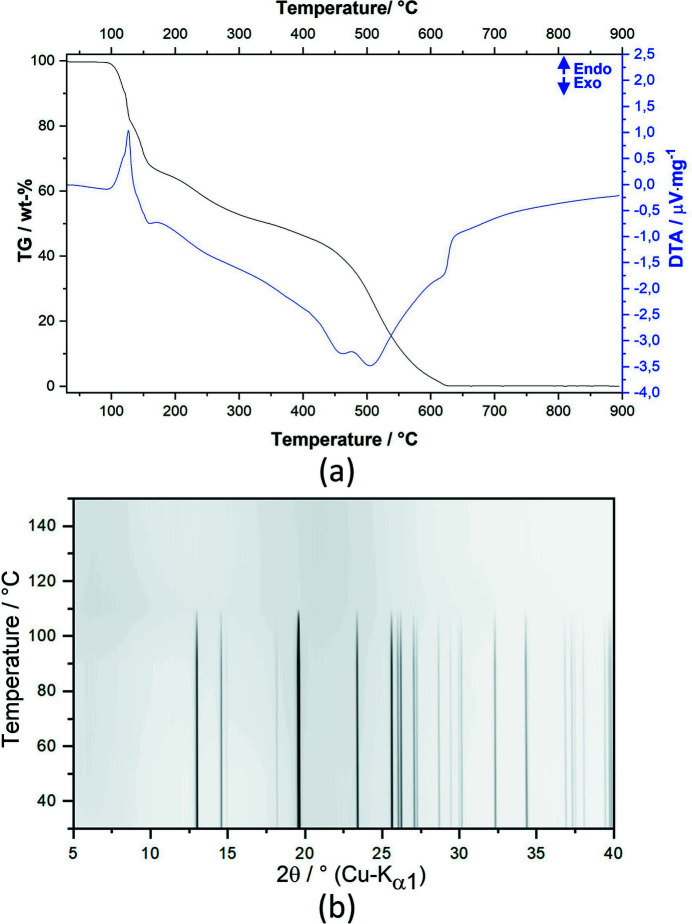
TG (black) and DTA (blue) curves for (1) measured in dynamic oxygen atmosphere (*a*). Temperature-dependent XRPD measurements of (1) showing the thermal stability up to *ca* 100°C (*b*).

Thermal analysis (TG and DTA) was performed to study the thermal behavior of compound (1) [Fig. 8 ▸(*a*)]. Several events related to mass losses are visible, but they are overlapped, making the separation of the different contributions difficult. The TG-curve showed that the sample is stable up to 100°C. Only a tiny weight loss is observed at the beginning that can be attributed to adsorbed moisture. Starting at 100°C, weight losses are observed and can be associated with endothermic peaks followed by broad exothermic peaks that likely correspond to the melting and decomposition of the sample. The sample is completely decomposed at 650°C, and no residue is observed at the end of the measurement due to the oxygen atmosphere. The compound is also unstable after a certain period of time (around 1–2 weeks) at room temperature, turning yellow and then black.

Temperature-dependent XRPD measurements [Fig. 8 ▸(*b*)] confirmed the disappearance of the Bragg peaks where the sample melts and begins to decompose. No other crystalline phases are observed on further heating, suggesting that the decomposition event on heating occurs in the liquid state. Sublimation is not completely ruled out, although a sequence of discoloration was observed to be similar to the decomposition in the solid state after long times at ambient conditions.

## 4. Conclusions

We have determined the local and crystallographic structure and thermal properties of a novel, small, cyclic organic compound: 1,3,5-triazine-2,4,6-triyl)trimethanol. The molecular structure was deduced by combination of elemental analysis, NMR, and IR/Raman spectroscopy. The combination of PDF analysis as a fingerprint of the local, intramolecular structure allowed for less ambiguous distinction and ranking between the different molecular structure candidates and also enabled the determination of a most likely conformation. The crystal structure solution from laboratory XRPD data then confirmed the correct molecular composition and form and revealed a columnar arrangement where the molecules interact through inter- and intracolumnar hydrogen bonding networks. PDF refinements of the local structure model then suggested that localized distortions from the average crystal structure exist in the form of small translations and tilts of hydrogen-bonded stack segments. Rietveld and PDF co-refinement allowed for extracting a robust, symmetry-independent model with accurate unit-cell parameters and bond lengths and angles. Temperature-dependent XRPD measurements and thermal analysis (TGA and DTA) revealed that the compound rapidly degrades above 100°C.

In conclusion, this work demonstrates that PDF analysis can be a useful technique for resolving ambiguities in small molecule structure determination and give additional insights into ranking more and less likely conformations. In addition, it can improve small molecule crystal structure refinements by providing a direct constraint on bond lengths, and in understanding the type and magnitude of deviations of molecular orientation from the average structure.

## Supplementary Material

Crystal structure: contains datablock(s) I. DOI: 10.1107/S2052520621010088/yb5032sup1.cif

Rietveld powder data: contains datablock(s) profile1. DOI: 10.1107/S2052520621010088/yb5032Isup2.rtv

NMR spectra, further information on PDF analysis, expanded plots for PDF/Rietveld refinements, overlay of structure models. DOI: 10.1107/S2052520621010088/yb5032sup3.pdf

CCDC reference: 2061886

## Figures and Tables

**Table 1:** Assignment of the IR and Raman bands for (1) vs: very strong, s: strong, m: medium, br: broad, vbr: very broad, sh: shoulder, w: weak.

Position (cm^−1^), shape		
IR	Raman	Assignment
3290, s	3292, vs	OH/NH stretching, NH stretching
–	3153, vbr	OH stretching/water, NH stretching
3129, vbr	–	OH stretching, NH stretching
–	2969, sh	CH stretching
2949, w	2945, sh	CH/CH_2_ stretching
–	2934, vs	CH stretching
2918, w	2920, sh	CH_2_sym/CH_2_asym stretching, CH stretching
	2871, m	CH/CH_2_ stretching
2857, br	–	CH stretching
2784, br	–	
–	2727, br	
2703, br	2705, sh	CH_2_ bending
2590, br		
1482, w	1480, m	CH_2_ bending, CH_2_ bending
1456, sh	1462, sh	
1440, s	1445, m	CH_2_ bending, CH_2_ bending
1403, w	–	
–	1375, m	NH/CH_2_ bending or CN stretching
1365, m	1362, m	
1338, m	1339, m	HCCN/*R*_sym_/CCH bending,
		HCCN/ring/CCH bending
1285, w	–	
1266, m	1262, s	COH/CH_2_ bending, COH/CH_2_ bending
1258, sh	–	
1174, m	1172, s	
1133, s	1132, sh	CC/CN stretching, CH bending
1122, s		
1103 vs	1105, s	CC/CO stretching, CH_2_/HCCN bending, CC/CN/CO stretching or CH_2_/ring bending
1073, m	–	CO stretching, *R* deformation
1040, vs	1040, w	CH bending, *R* deformation, CN stretching, CN stretching
959, s	960, s	C—OH stretching
910, vs	913, m	CC stretching, CH_2_/*R*_sym_ bending
–	843, sh	CNCC/*R*_sym_/CH_2_ bending
843, sh	829, m	CH/CNCC/*R*_asym_/CH_2_ bending, NH bending
734, br	–	N—H wagging
690, s		
674, sh		
624, s		
617, sh		
490, vs	493, s	Lattice modes
435, w	–	
	354, m	
	327, w	
	281, w	
	221, w	

**Table 2:** Crystallographic details and parameter values obtained from Rietveld refinement for 1

Compound	Glycolaldehyde ammonia
Molecular formula	C_6_H_12_N_3_O_3_
Molecular weight (g mol^−1^)	174.19
Crystal system	Orthorhombic
Space group	*Ama*2
Wavelength (Å)	1.5406
*a*, *b*, *c* (Å)	12.1054 (2), 13.5537 (2), 5.20741 (8)
*V* (Å^3^)	854.40 (2)
*T* (K)	298
No. refined parameters	34
*D*_calc_ (g cm^−3^)	1.35
*R*_wp_ (%)	6.51
*R*_p_ (%)†	4.63
*R*_Bragg_ (%)†	5.30
Starting angle measured, 2θ (°)	0
Final angle measured, 2θ (°)	110
Starting angle used, 2θ (°)	5
Final angle used, 2θ (°)	100
Step width, 2θ (°)	0.01
Time (h)	12

† As defined in *TOPAS 6.0*.

**Table 3:** Model parameter values obtained by co-refinement to the XRPD/PDF data of (1)

Crystal system	Triclinic
Space group	*P*1
*a*,*b*,*c* (Å)	13.5545 (1), 5.20787 (5), 12.1060 (1)
α, β, γ (°)	90.027 (3), 90.022 (4), 89.970 (4)
*V* (Å^3^)	854.58 (1)
*R*_wp_ (%) for XRPD†	5.28
*R*_wp_ (%) for PDF†	15.8

† As defined in *TOPAS 6.0*.

## References

[bb1] ACD (2019). *ACD*/*NMR Suite.* Advanced Chemistry Development, Inc., Toronto, ON, Canada. www.acdlabs.com.

[bb2] Aschan, O. (1915). *Ber. Dtsch. Chem. Ges.* **48**, 874–891.

[bb3] Baur, E. & Rüetschi, W. (1941). *Helv. Chim. Acta*, **24**, 754–767.

[bb4] Benmore, C. J., Weber, J. K. R., Tailor, A. N., Cherry, B. R., Yarger, J. L., Mou, Q., Weber, W., Neuefeind, J. & Byrn, S. R. (2013). *J. Pharm. Sci.* **102**, 1290–1300. 10.1002/jps.23464 23381910

[bb5] Billinge, S. J. L., Dykhne, T., Juhás, P., Božin, E., Taylor, R., Florence, A. J., Shankland, K. (2010). *CrystEngComm*, **12**, 1366–1368.

[bb6] Bryce, D. L., Bernard, G. M., Gee, M., Lumsden, M. D., Eichele, K. & Wasylishen, R. E. (2001). *Can. J. Anal. Sci. Spectrosc.* **46**, 46–82.

[bb7] Butlerow, A. (1860). *Ann. Chem. Pharm.* **115**, 322–327.

[bb8] Chan, F. C., Anwar, J., Cernik, R., Barnes, P. & Wilson, R. M. (1999). *J. Appl. Cryst.* **32**, 436–441.

[bb9] Cheary, R. W., Coelho, A. A. & Cline, J. P. (2004). *J. Res. NIST*, **109**, 1–25. 10.6028/jres.109.002 PMC4849620 27366594

[bb10] Chupas, P. J., Qiu, X., Hanson, J. C., Lee, P. L., Grey, C. P. & Billinge, S. J. L. (2003). *J. Appl. Cryst.* **36**, 1342–1347.

[bb11] Clark, S. J., Segall, M. D., Pickard, C. J., Hasnip, P. J., Probert, M. I. J., Refson, K. & Payne, M. C. (2005). *Z. Kristallogr. Cryst. Mater.* **220**, 567.

[bb15] Coelho, A. A. (2000). *J. Appl. Cryst.* **33**, 899–908.

[bb13] Coelho, A. A. (2003). *J. Appl. Cryst.* **36**, 86–95.

[bb12] Coelho, A. A., Chater, P. A. & Kern, A. (2015). *J. Appl. Cryst.* **48**, 869–875.

[bb14] Coelho, A. A. (2017). *Topas 6.0.* Bruker AXS, Karlsruhe, Germany.

[bb100] Dassault Systèmes (2018). *Materials Studio*. BIOVIA, Dassault Systemes, San Diego, California, USA

[bb16] Dwarakanath, K. & Sathyanarayana, D. N. (1979). *Bull. Chem. Soc. Jpn*, **52**, 2699–2704.

[bb200] Ernst, M., Huber, T., Melder J.-P., Jaegli, S. & Krug, T. (2020). Patent WO/2020/249428. BASF SE, Germany.

[bb32] Fox, M. A. & Whitesell, J. K. (1995). *Organische Chemie*: *Grundlagen, Mechanismen, Bioorganische Anwendungen*. Verlag: Spektrum Akademischer Verlag

[bb17] George, C. & Gilardi, R. (1987). *Acta Cryst.* C**43**, 1003–1005.

[bb18] Ghandi, M., Salimi, F. & Olyaei, A. (2006). *Molecules*, **11**, 556–563. 10.3390/11070556 PMC6148636 17971727

[bb19] Giumanini, A. G., Verardo, G., Randaccio, L., Bresciani-Pahor, N. & Traldi, P. (1985). *J. Prakt. Chem.* **327**, 739–748.

[bb20] Halgren, T. A. (1999). *J. Comput. Chem.* **20**, 720–729. 10.1002/(SICI)1096-987X(199905)20:7<720::AID-JCC7>3.0.CO;2-X 34376030

[bb21] Hammersley, A. P., Svensson, S. O., Hanfland, M., Fitch, A. N. & Hausermann, D. (1996). *High Press. Res.* **14**, 235–248.

[bb22] Hirokawa, T., Kimura, T., Ohno, K. & Murata, M. (1980). *Spectrochim. Acta A*, **36**, 329–332.

[bb23] Isac Paulraj, E. & Muthu, S. (2013). *Spectrochim. Acta A Mol. Biomol. Spectrosc.* **108**, 38–49. 10.1016/j.saa.2013.01.061 23454843

[bb24] Juhás, P., Cherba, D. M., Duxbury, P. M., Punch, W. F. & Billinge, S. J. L. (2006). *Nature*, **440**, 655–658. 10.1038/nature04556 16572167

[bb25] Juhás, P., Davis, T., Farrow, C. L. & Billinge, S. J. L. (2013). *J. Appl. Cryst.* **46**, 560–566.

[bb26] Juhás, P., Farrow, C., Yang, X., Knox, K. & Billinge, S. (2015). *Acta Cryst.* A**71**, 562–568. 10.1107/S2053273315014473 26522405

[bb27] Kitagawa, S., Yokoi, T. & Kaito, M. (1987). Patent No. 4677213. Research Association for Utilization of Light Oil, Tokyo, Japan.

[bb28] Kobayashi, Y., Takahara, H., Takahashi, H. & Higasi, K. (1976). *J. Mol. Struct.* **32**, 235–246.

[bb29] Lund, E. W. (1951). *Acta Chem. Scand.* **5**, 678–680.

[bb30] Macrae, C. F., Sovago, I., Cottrell, S. J., Galek, P. T. A., McCabe, P., Pidcock, E., Platings, M., Shields, G. P., Stevens, J. S., Towler, M. & Wood, P. A. (2020). *J. Appl. Cryst.* **53**, 226–235. 10.1107/S1600576719014092 PMC6998782 32047413

[bb31] Mallo, N., Foley, E. D., Iranmanesh, H., Kennedy, A. D. W., Luis, E. T., Ho, J., Harper, J. B. & Beves, J. E. (2018). *Chem. Sci.* **9**, 8242–8252. 10.1039/c8sc03218a PMC6240811 30542573

[bb33] McMurry, J. E. (1992). *Organic Chemistry*, 3rd ed. Belmont, CA: Brooks Cole.

[bb34] Mou, Q., Benmore, C. J. & Yarger, J. L. (2015). *J. Appl. Cryst.* **48**, 950–952.

[bb35] Neelakantan, P. (1963). *Proc. Indian Acad. Sci.* **57**, 94–102.

[bb36] Nielsen, A. T., Atkins, R. L., DiPol, J. & Moore, D. W. (1974). *J. Org. Chem.* **39**, 1349–1355.

[bb37] Nielsen, A. T., Atkins, R. L., Moore, D. W., Scott, R., Mallory, D. & LaBerge, J. M. J. T. (1973) *J. Org. Chem.* *38*, 3288–3295.

[bb38] Nielsen, A. T., Moore, D. W., Ogan, M. D. & Atkins, R. L. (1979). *J. Org. Chem.* **44**, 1678–1684.

[bb39] Pagola, S., Stephens, P. W., He, X. & Byrn, S. R. (2001). *Mater. Sci. Forum*, **378–381**, 789–794.

[bb40] Pawley, G. S. (1981). *J. Appl. Cryst.* **14**, 357–361.

[bb42] Peterson, P. F., Božin, E. S., Proffen, Th. & Billinge, S. J. L. (2003). *J. Appl. Cryst.* **36**, 53–64.

[bb43] Prill, D., Juhás, P., Billinge, S. J. L. & Schmidt, M. U. (2016). *Acta Cryst.* A**72**, 62–72. 10.1107/S2053273315022457 26697868

[bb44] Prill, D., Juhás, P., Schmidt, M. U. & Billinge, S. J. L. (2015). *J. Appl. Cryst.* **48**, 171–178.

[bb45] Pütz, A. M., Terban, M. W., Bette, S., Haase, F., Dinnebier, R. E. & Lotsch, B. V. (2020). *Chem. Sci.* **11**, 12647–12654 . 10.1039/d0sc03048a PMC8163241 34094458

[bb46] Rademacher, N., Daemen, L. L., Chronister, E. L. & Proffen, Th. (2012). *J. Appl. Cryst.* **45**, 482–488.

[bb47] Rietveld, H. M. (1969). *J. Appl. Cryst.* **2**, 65–71.

[bb41] Roose, P., R., Eller, K., Henkes, E., Rossbacher, R. & Höke, H. (2015). *Ullmann’s Encyclopedia of Industrial Chemistry*. *Amines*, *Aliphatic.* Wiley VCH.

[bb48] Schlesinger, C., Habermehl, S. & Prill, D. (2021). *J. Appl. Cryst.* **54**, 776–786. 10.1107/S1600576721002569 PMC8202035 34188612

[bb49] Schlesinger, C., Hammer, S. M., Gorelik, T. E. & Schmidt, M. U. (2020). *Acta Cryst.* B**76**, 353–365. 10.1107/S2052520620003984 32831256

[bb50] Spek, A. L. (2009). *Acta Cryst.* D**65**, 148–155. 10.1107/S090744490804362X PMC2631630 19171970

[bb51] Sun, C., Zhu, J., Wang, H., Jin, J., Xing, J. & Yang, D. (2011). *Eur. J. Med. Chem.* **46**, 11–20. 10.1016/j.ejmech.2010.09.047 21093112

[bb52] Terban, M. W., Cheung, E. Y., Krolikowski, P. & Billinge, S. J. L. (2015). *Cryst. Growth Des.* **16**, 210–220.

[bb53] Terban, M. W., Russo, L., Pham, T. N., Barich, D. H., Sun, Y. T., Burke, M. D., Brum, J. & Billinge, S. J. L. (2020). *Mol. Pharm.* **17**, 2370–2389. 10.1021/acs.molpharmaceut.0c00122 32293895

[bb54] Thomas, S., Biswas, N., Venkateswaran, S., Kapoor, S., D’Cunha, R. & Mukherjee, T. (2005). *Chem. Phys. Lett.* **402**, 361–366.

[bb55] Tuguldurova, V. P., Fateev, A. V., Malkov, V. S., Poleshchuk, O. K., Vodyankina, O. V. (2017). *J. Phys. Chem. A*, **121**, 3136–3141. 10.1021/acs.jpca.7b00823 28380298

[bb56] Xue, S., Bu, H., Liu, L., Xu, X. & Ma, X. (2011). *Chin. J. Chem.* **29**, 1011–1016.

[bb57] Yang, X., Juhas, P., Farrow, C. L. & Billinge, S. J. (2014). arXiv:1402.3163.

[bb58] Yates, J. R., Pickard, C. J. & Mauri, F. (2007). *Phys. Rev. B*, **76**, 024401.

