# Supplementary material for: A previously unknown cyclic alkanolamine and molecular ranking using the pair distribution function
Source: Acta Crystallogr B Struct Sci Cryst Eng Mater. 2021 Nov 17;77(Pt 6):986–95. doi: 10.1107/S2052520621010088 (PMC8662943; doi:10.1107/S2052520621010088)
Supplement: Supplementary file 3 [file b-77-00986-sup3.pdf]

A previously unknown cyclic alkanolamine and molecular ranking using the pair distribution function

Gianpiero Gallo*[a,b], Maxwell W. Terban*[a], Igor Moudrakovski [a], Tatjana Huber[c], Martin Etter[d], Martin Ernst[c], Bernd Hinrichsen[c], Robert E. Dinnebier[a]

[a]Max Planck Institute for Solid State Research, Heisenbergstraße 1, 70569 Stuttgart, Germany
[b]Department of Chemistry and Biology “A. Zambelli”, University of Salerno, Via Giovanni Paolo II, 132, Fisciano (SA), 84084, Italy.
[c]BASF SE, Carl-Bosch-Strasse 38, 67056 Ludwigshafen am Rhein, Germany.
[d]Deutsches Elektronen-Synchrotron (DESY), Notkestrasse 85, 22607 Hamburg, Germany.

Additional tables and figures

Contents

1. NMR 2
2. Pair distribution function (PDF) 5
3. X-ray powder diffraction (XRPD) 12
4. XRPD/PDF co-refinement 16
References 16

1. NMR

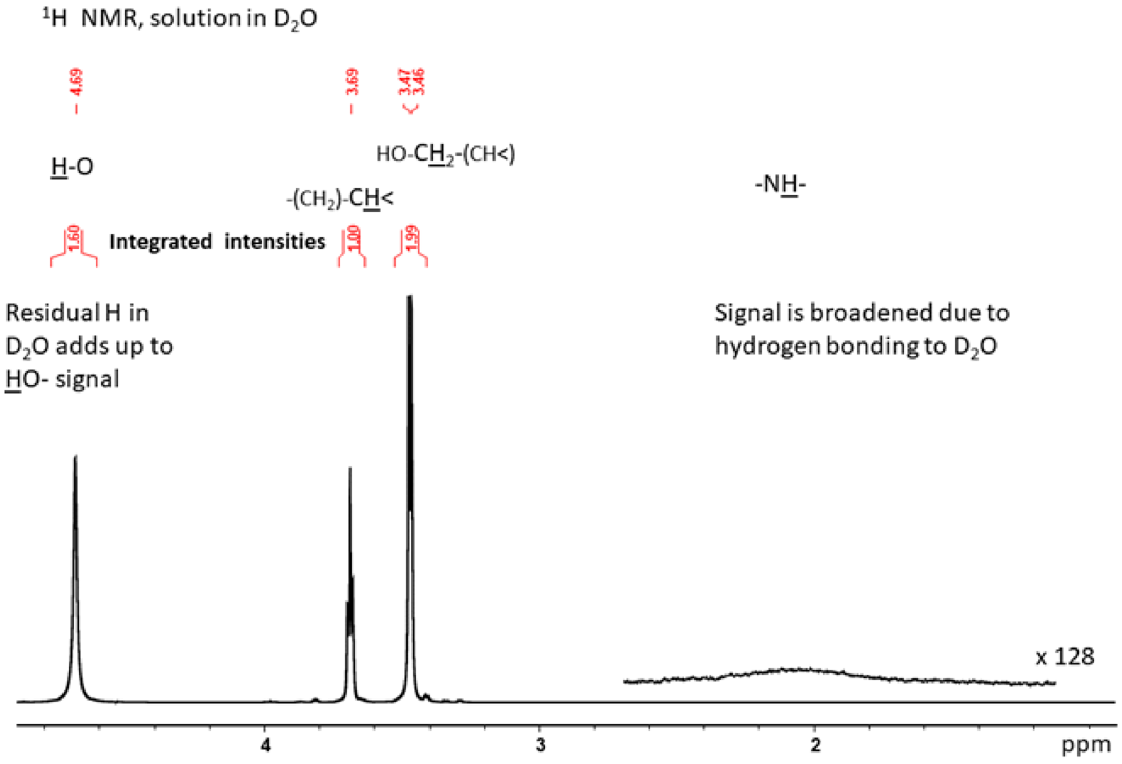

Figure S1. ^1H NMR spectrum of a solution of **1** in D_2O.

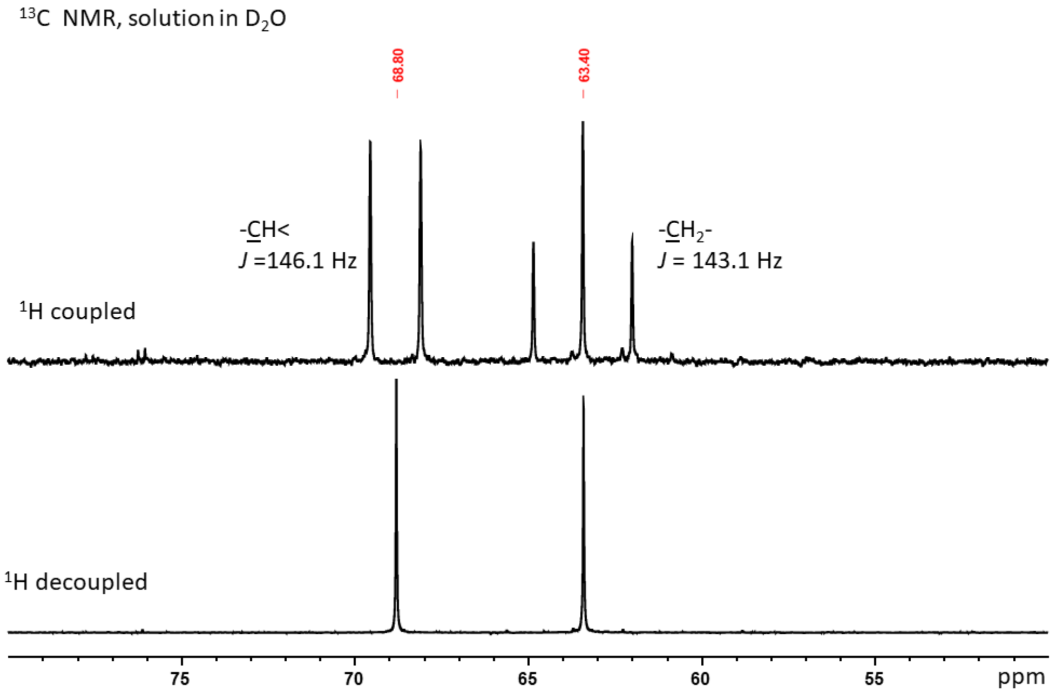

Figure S2. ^{13}C NMR spectra (^1H coupled, above and ^1H decoupled, below) of a solution of **1** in DMSO-d6.

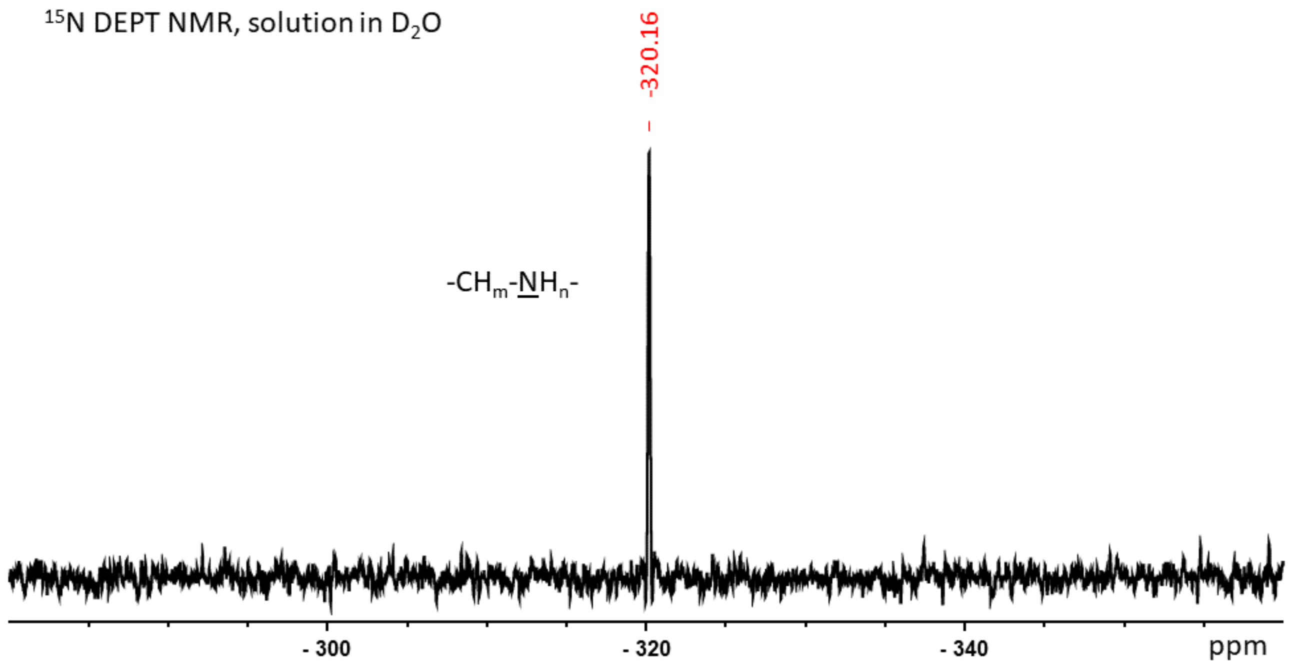

Figure S3. ^{15}N NMR spectrum of a solution of **1** in DMSO-d6.

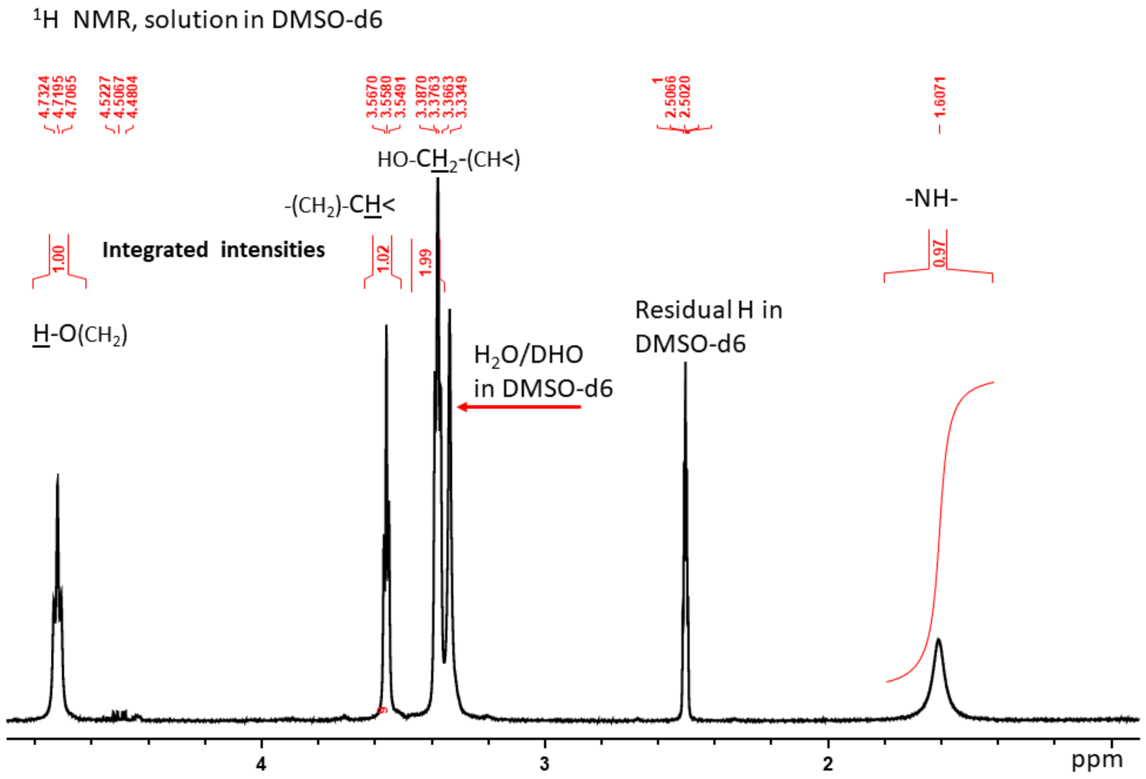

Figure S4. ^{1}H NMR spectrum of a solution of **1** in DMSO-d6.

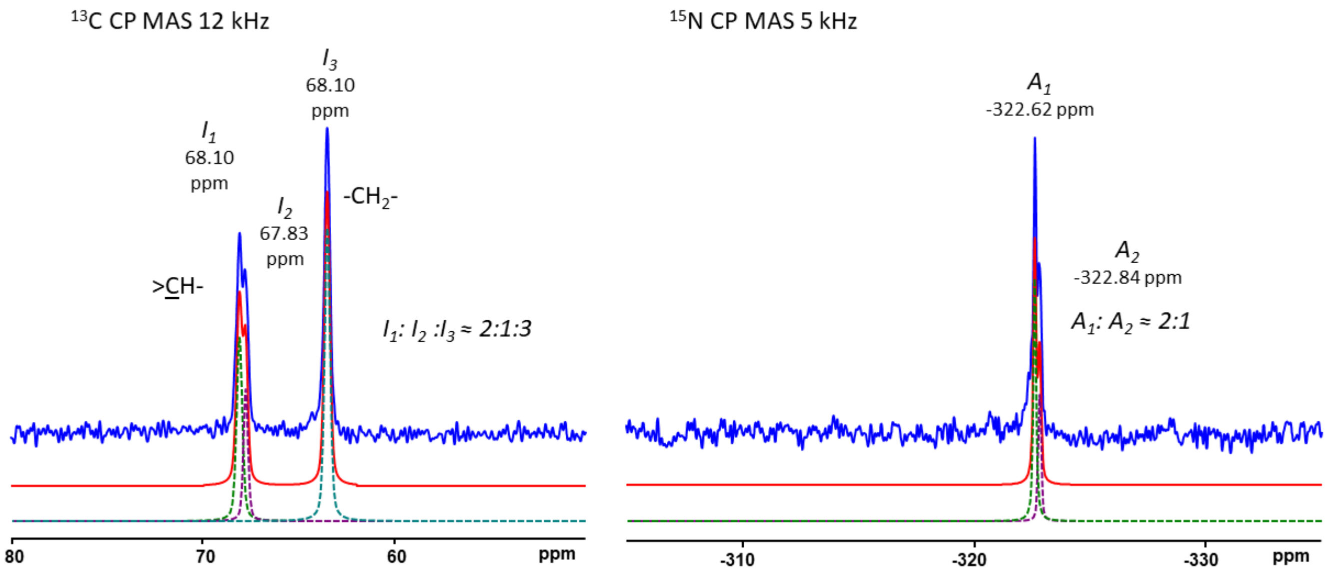

Figure S5. ^{13}C (right) and ^{15}N (left) CP MAS solid-state NMR spectra of **1**.

2. Pair distribution function (PDF)

Total scattering measurements over a wide range of momentum transfer and with good statistics are required to obtain suitable PDFs for structure analysis. The coherent powder diffraction intensities $I_c(Q)$ are normalized by the form factors of the sample composition to obtain the total scattering structure function $S(Q)$ by

$$S(Q) = \frac{I_c(Q) - \langle f(Q)^2 \rangle + \langle f(Q) \rangle^2}{\langle f(Q) \rangle^2}, \tag{1}$$

which is then Fourier transformed by

$$G(r) = \frac{2}{\pi} \int_{Q_{min}}^{Q_{max}} Q[S(Q) - 1] sin(Qr) dQ. \tag{2}$$

$F(Q) = Q[S(Q) - 1]$ is the reduced total scattering structure function, and $G(r)$ is the PDF. In practice, values of Q_{min} and Q_{max} are determined by the experimental setup, and Q_{max} is often reduced below the experimental maximum to reduce the effects of low signal-to-noise in the high-Q region on the Fourier transformation. The PDF gives the scaled probability of finding two atoms in a material a distance r apart and is relative to the density of atom pairs in the material. $G(r)$ can be calculated from a known structure model according to

$$G(r) = \frac{1}{\mathrm{r}N} \left(\sum_i \sum_{j \neq i} \frac{f_i f_j}{\langle f \rangle^2} \delta(r - r_{ij}) \right) - 4\pi r \rho_0. \tag{3}$$

Here, ρ_0 is the average number density of the material, and the sums run over all atoms in the sample; f_i is the scattering factor of atom i, $\langle f \rangle$ is the average scattering factor, and r_{ij} is the distance between atoms i and j. Refinement and fitting of crystallographic structures to the PDF data were performed using TOPAS v6[1].

$F(Q)$ for discrete molecules was calculated using the Debye function as implemented in Diffpy-CMI[2], wherein

$$F(Q) = \frac{1}{N} \sum_i \sum_{j \neq i} \frac{f_i f_j}{\langle f \rangle^2} \frac{sin(Qr)}{r}. \tag{4}$$

The calculations were performed with the atomic displacement parameter $B_{iso} = 0.2$ Å^2 for every atom, $Q_{damp} = 0.03$ Å^{-1} and $Q_{broad} = 0.01$ Å^{-1}. $G(r)$ was subsequently obtained by inputting this into Eq. 2 with $Q_{min} = 1.5$ Å^{-1} and $Q_{max} = 22$ Å^{-1}. For comparison and ranking, the Pearson product-momentum correlation coefficient (PCC) is calculated by

$$\mathrm{PCC} = \frac{1}{n-1}\sum_{i=0}^{n}\left(\frac{X_i - \bar{X}}{\sigma_x}\right)\left(\frac{Y_i - \bar{Y}}{\sigma_y}\right) \quad (5)$$

Where $\bar{X}$ and $\bar{Y}$ are the means and σ_x and σ_y are the standard deviations of the respective datasets. This calculation gives a value between −1 and 1 where −1 implies anticorrelation, 0 implies no correlation, and 1 implies perfect correlation.

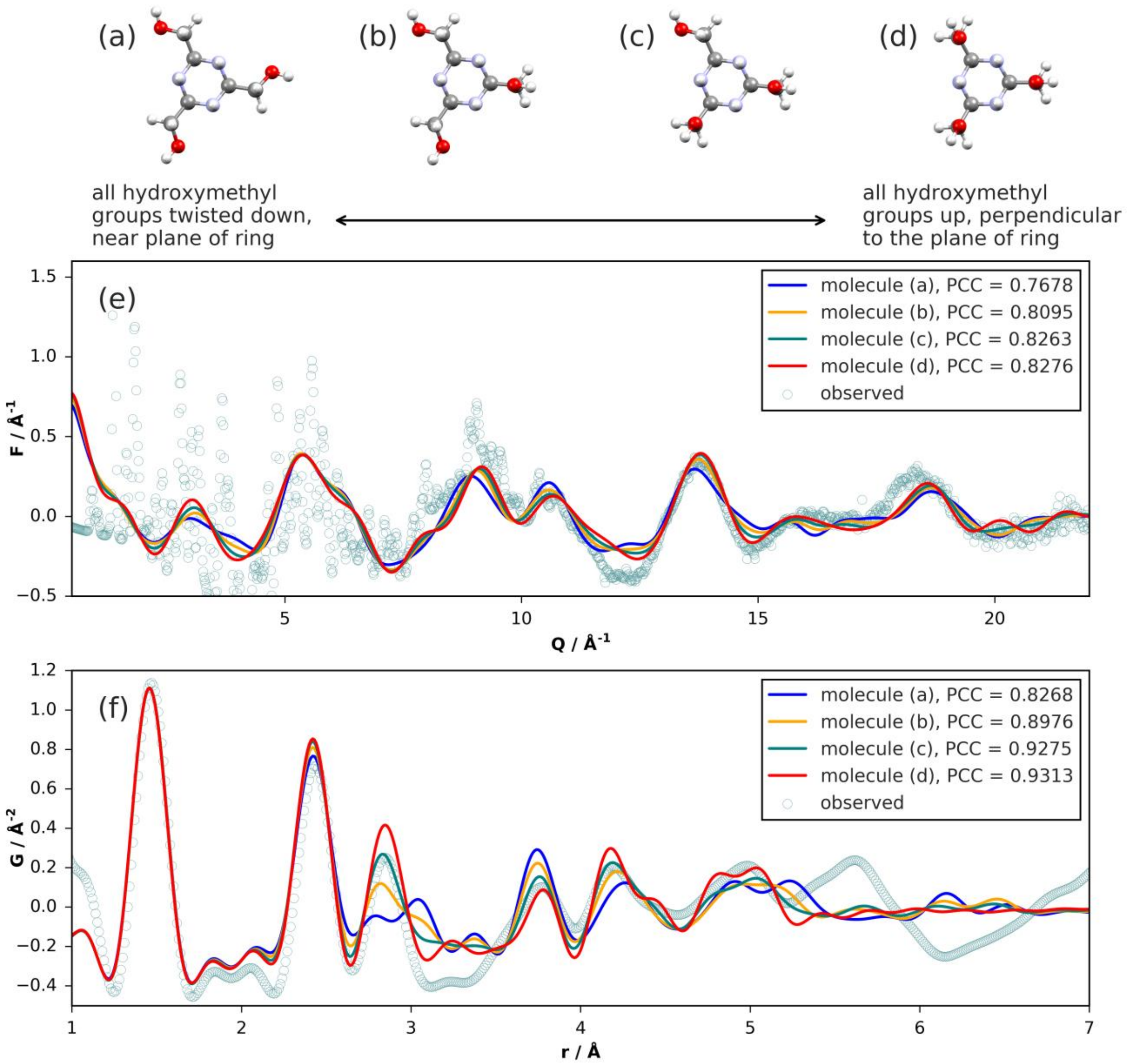

Figure S6. Four representative conformations of the best molecular structure investigated (a-d) looking along the plane of the ring. Comparisons of simulated and experimental *F*(*Q*) and *G*(*r*) and PCC values (compared over ranges of *Q* = 6.0–22.0 Å^{-1} and *r* = 1.9–5.25 Å) are given in (e) and (f) respectively. Conformation (a) has all hydroxymethyl groups twisted down, pointing close to but just out of the plane of the ring. Molecules (b-d) represent conformations with one, two, or all three hydroxymethyl groups flipped upward, pointing perpendicular to the plane of the ring, respectively.

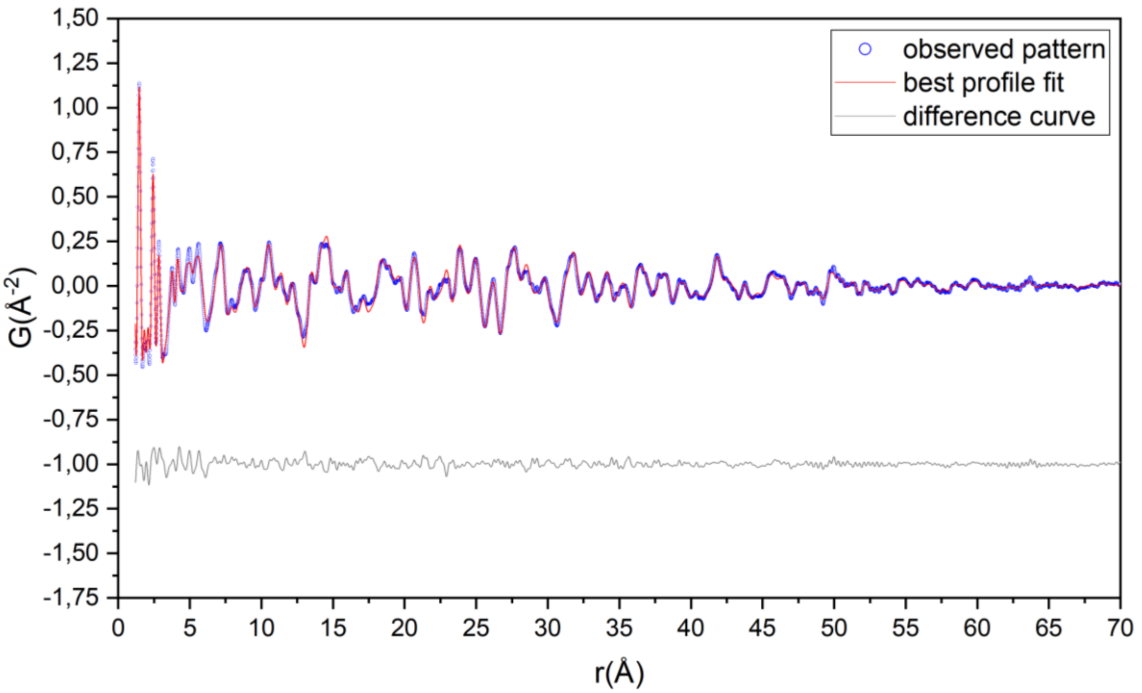

Figure S7. PDF fit of the model with *Ama*2 space group (model 1) in a '*r*'-range from 1.2 to 70 Å. The blue circles and red solid line correspond to measured and simulated PDFs, respectively. The grey solid line offset below denotes the difference curve.

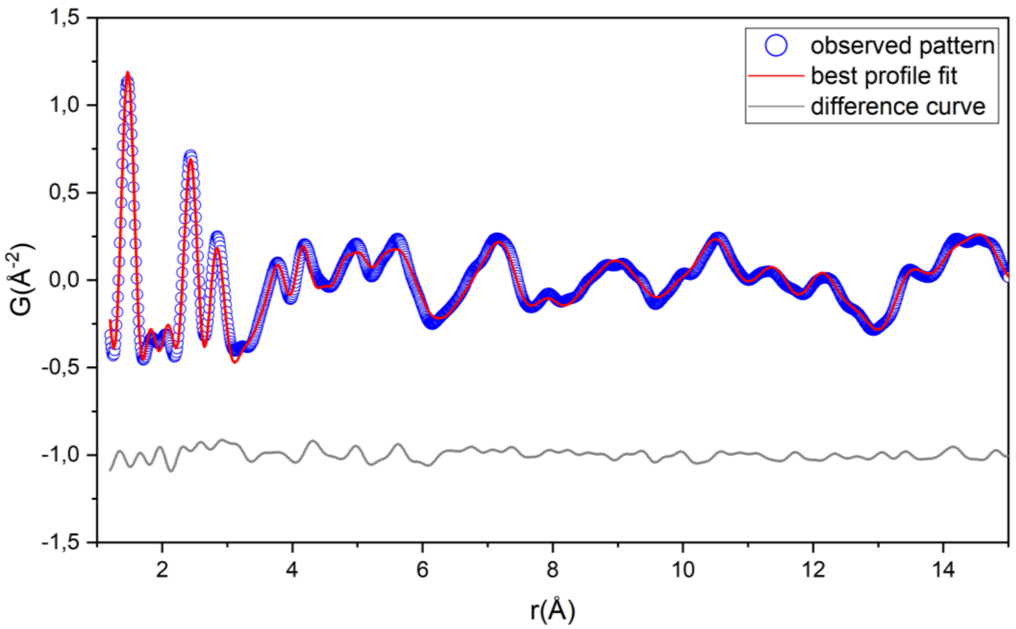

Figure S8. PDF fit of the model with *Ama*2 space group (model 1) in a '*r*'-range from 1.2 to 15 Å. The blue circles and red solid line correspond to measured and simulated PDFs, respectively. The grey solid line offset below denotes the difference curve.

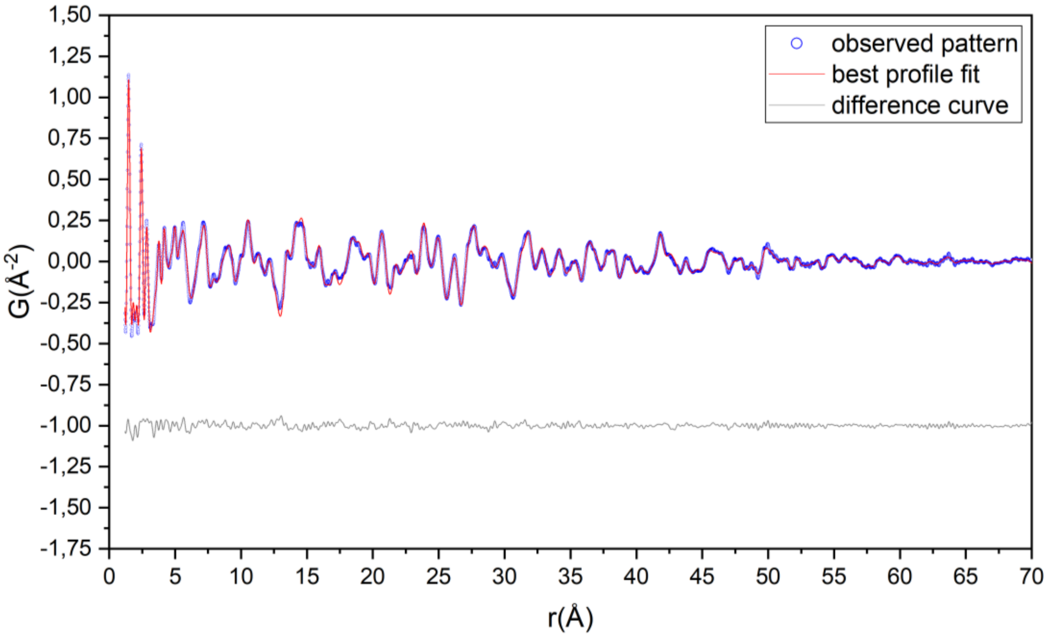

Figure S9. PDF fit of the model with *C*2 space group (model 2) in a '*r*'-range from 1.2 to 70 Å. The blue circles and red solid line correspond to measured and simulated PDFs, respectively. The grey solid line offset below denotes the difference curve.

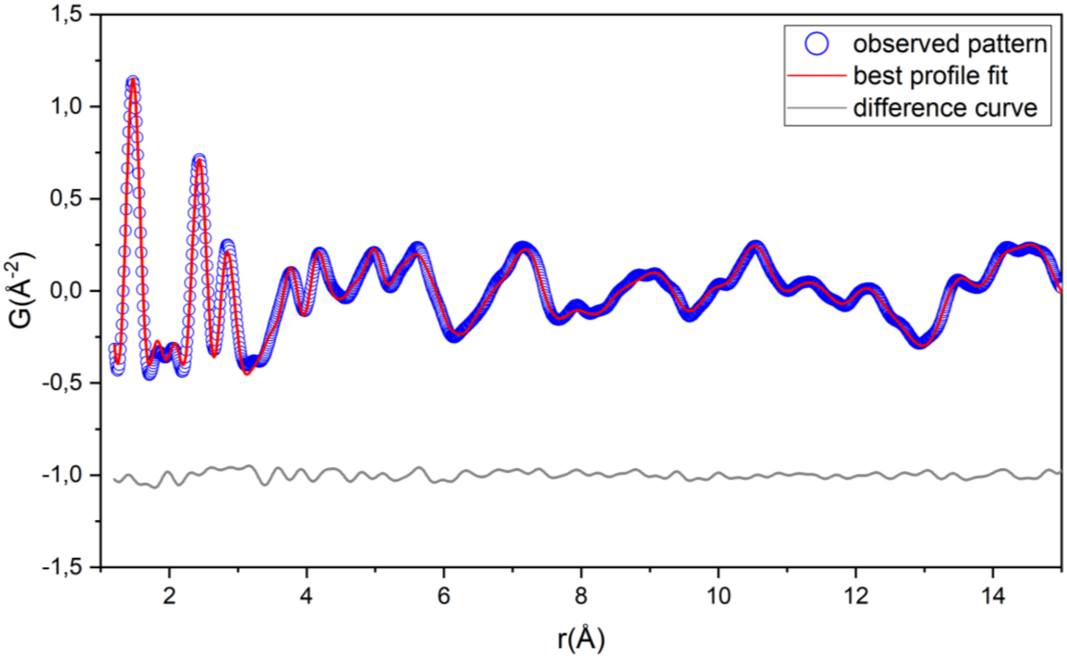

Figure S10. PDF fit of the model with *C*2 space group (model 2) in a '*r*'-range from 1.2 to 15 Å. The blue circles and red solid line correspond to measured and simulated PDFs, respectively. The grey solid line offset below denotes the difference curve.

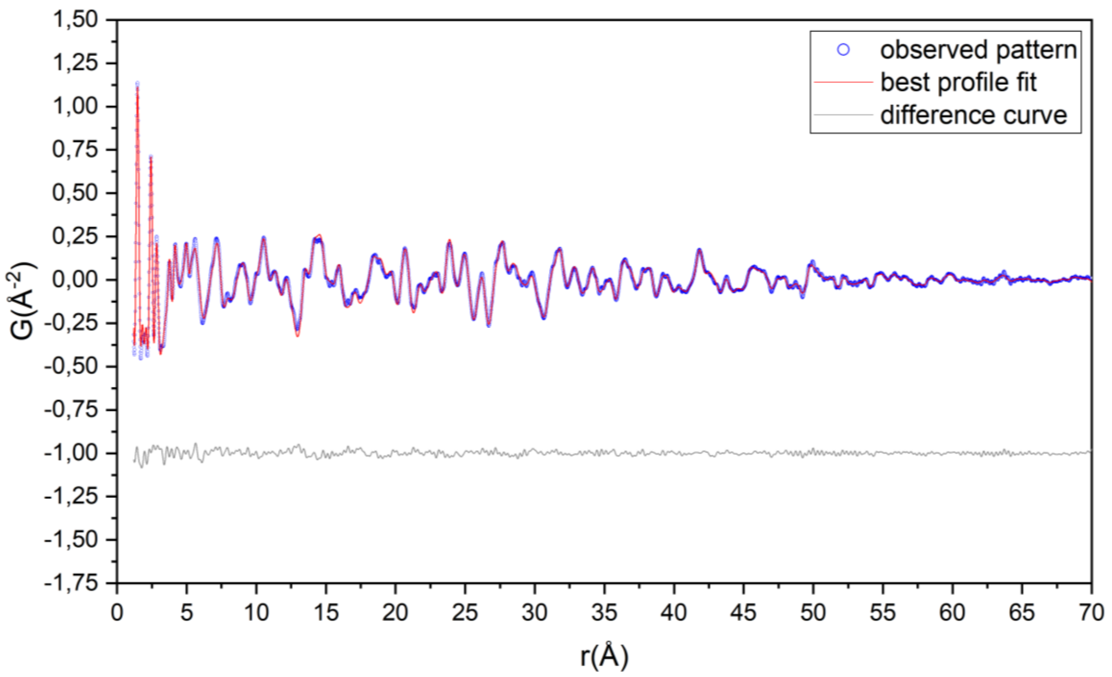

Figure S11. PDF fit of the model with *P*1 space group (model 3) in a '*r*'-range from 1.2 to 70 Å. The blue circles and red solid line correspond to measured and simulated PDFs, respectively. The grey solid line offset below denotes the difference curve.

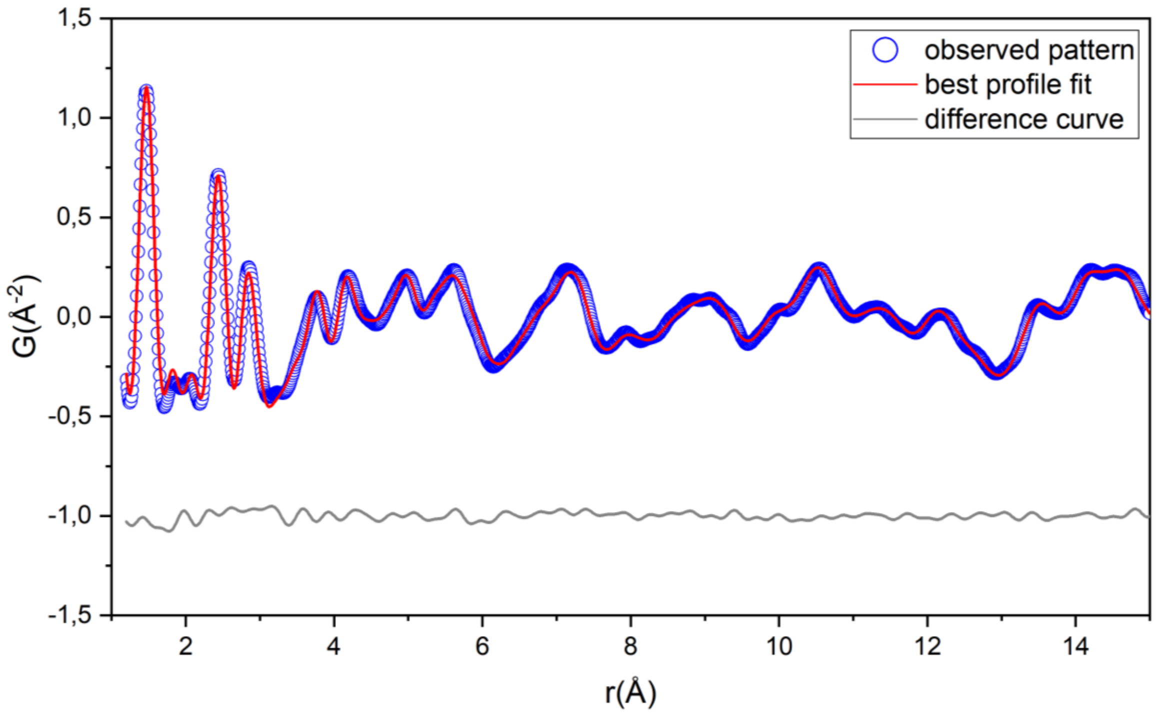

Figure S12. PDF fit of the model with *P*1 space group (model 3) in a '*r*'-range from 1.2 to 15 Å. The blue circles and red solid line correspond to measured and simulated PDFs, respectively. The grey solid line offset below denotes the difference curve.

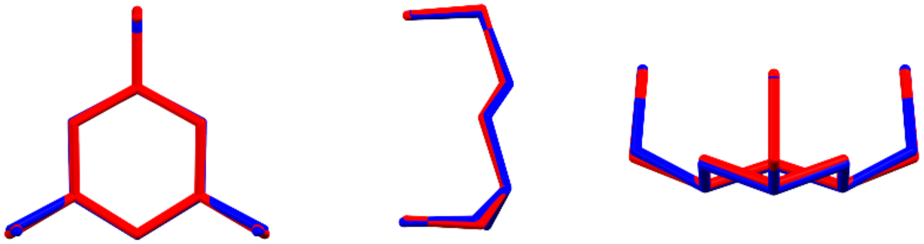

(a) (b) (c)

Figure S13. Superimposition of the molecules obtained from XRPD (red) and from PDF using the model *Ama*2 (blue). Root-mean-square deviation (RMSD): 0.738**.**

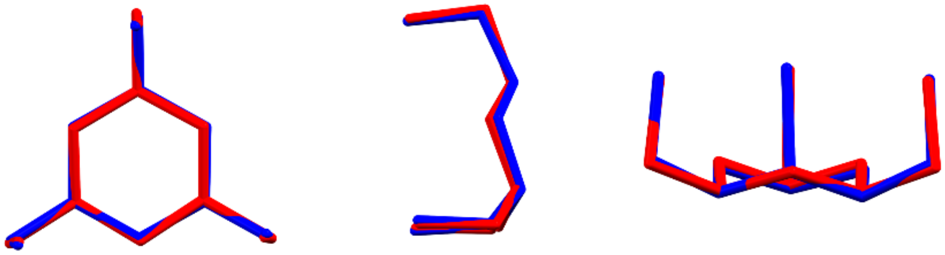

(a) (b) (c)

Figure S14. Superimposition of the molecules obtained from XRPD (red) and from PDF using the model *C*2 (blue). RMSD: 0.805**.**

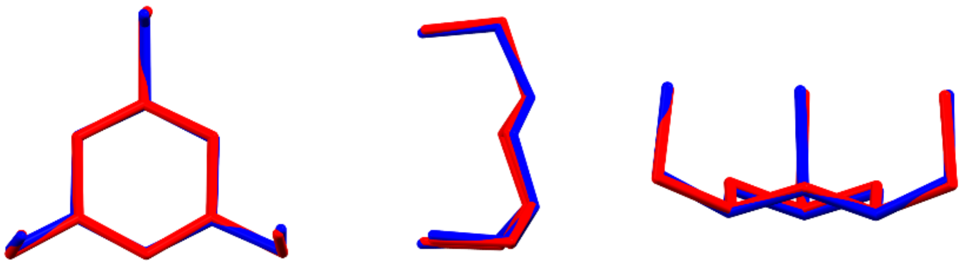

(a) (b) (c)

Figure S15. Superimposition of the molecules obtained from XRPD (red) and from PDF using the model *P*1 (blue). RMSD: 0.868**.**

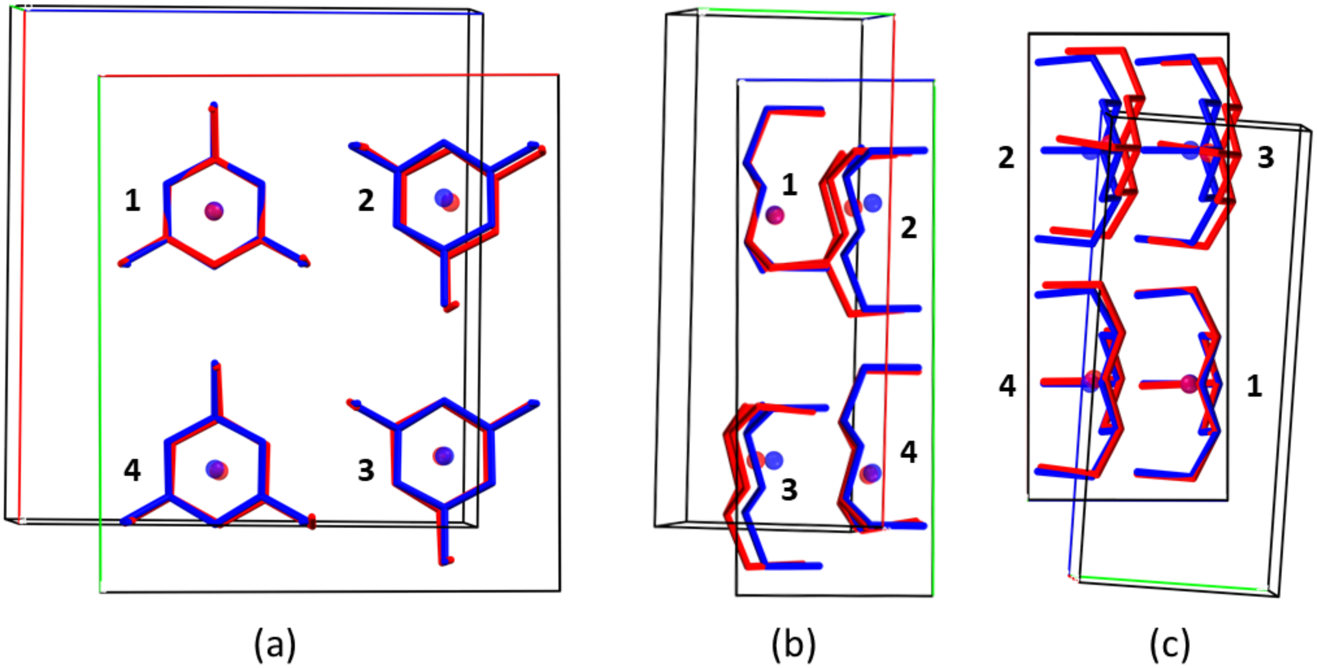

Figure S16. Comparison between the unit cells contents obtained from PDF refinements of the structure models with space groups *Ama*2 (blue) and *P*1 (red) in the range 1.2 – 15 Å. Non-H atoms of the molecules 1 were superimposed in order to show the difference between the two models. Distances between centroids, calculated using all non-H atoms, are 0.555 Å in **2**, 0.482 Å in **3**, and 0.149 Å in **4**. Centroids related to the models with space groups *Ama*2 and *P*1 are depicted in blue and red, respectively. Hydrogen atoms have been omitted for clarity.

Table S1. Crystallographic and PDF Refinement data of 1.

**Compound**	**Model 1**		**Model 2**		**Model 3**	
**Crystal system**	Orthorhombic		Monoclinic		Triclinic	
**Space group**	*Ama*2		*C*2		*P*1	
***a* / Å**	12.1096(9)	12.114(5)	13.5582(8)	13.567(6)	13.5580(9)	13.565(8)
***b*/ Å**	13.558(1)	13.637(3)	5.2091(3)	5.201(2)	5.2080(3)	5.207(3)
***c*/ Å**	5.2087(4)	5.197(3)	12.1153(7)	12.140(4)	12.1126(7)	12.120(6)
***α*/ °**	90	90	90	90	89.94(4)	90.1(3)
***β*/ °**	90	90	90.00(3)	90.3(2)	90.03(3)	90.1(3)
***γ*/ °**	90	90	90	90	90.08(3)	90.2(3)
**V/ Å^3**	855.2(1)	858.6(7)	855.65(9)	856.7(6)	855.3(1)	856.0(8)
**T/ K**	298	298	298	298	298	298
**refined parameters**	16		24		47	
**R_{wp}/% [a]**	17.9	14.6	13.3	10.3	12.9	9.8
**Starting ‘*r*’-range used/ Å**	1.2	1.2	1.2	1.2	1.2	1.2
**Final ‘*r*’-range used/ ° Å**	70	15	70	15	70	15

[a]as defined in TOPAS 4.

3. X-ray powder diffraction (XRPD)

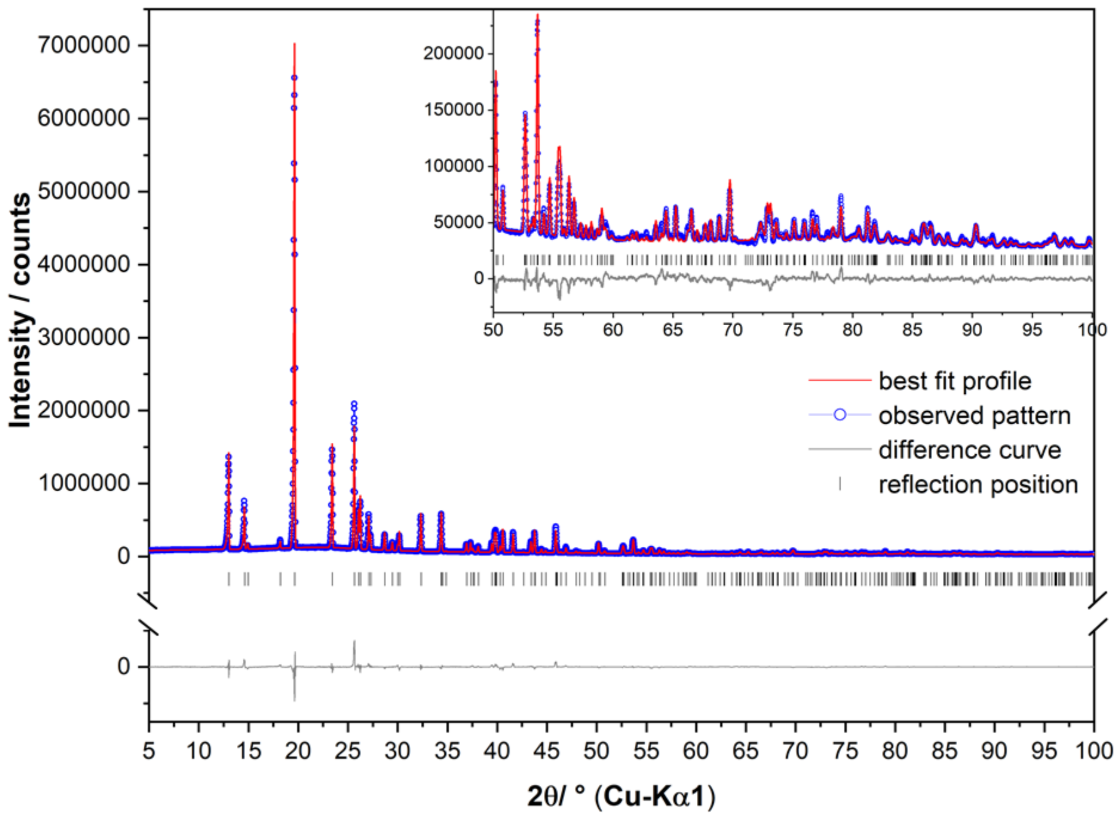

Figure S17. Rietveld plot of the model with *Ama*2 space group obtained from PDF refinement. The observed pattern (circles), the best Rietveld fit profile (line) and the difference curve between the observed and the calculated profiles (below) are shown. The high angle part starting at 50° in 2θ is enlarged for clarity.

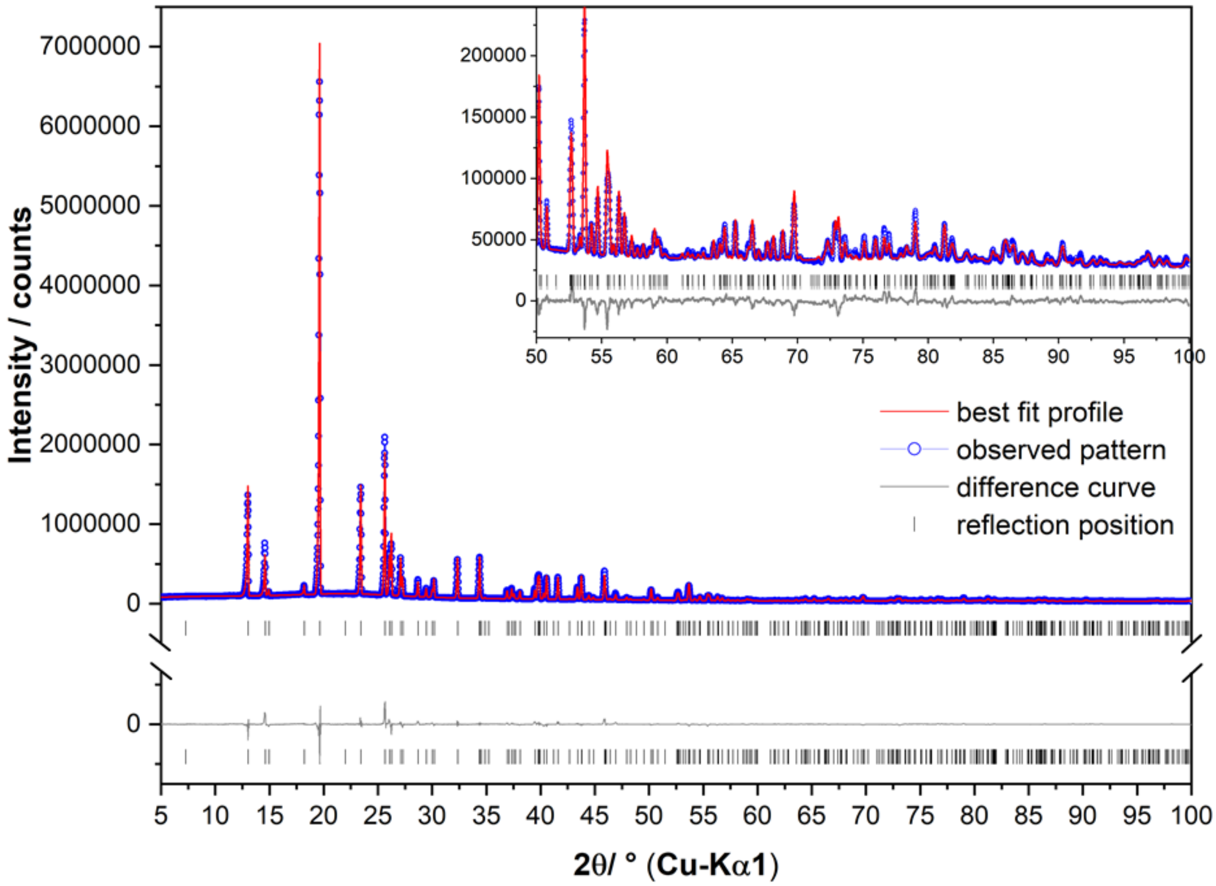

Figure S18. Rietveld plot of the model with *C*2 space group obtained from PDF refinement. The observed pattern (circles), the best Rietveld fit profile (line) and the difference curve between the observed and the calculated profiles (below) are shown. The high angle part starting at 50° in 2θ is enlarged for clarity.

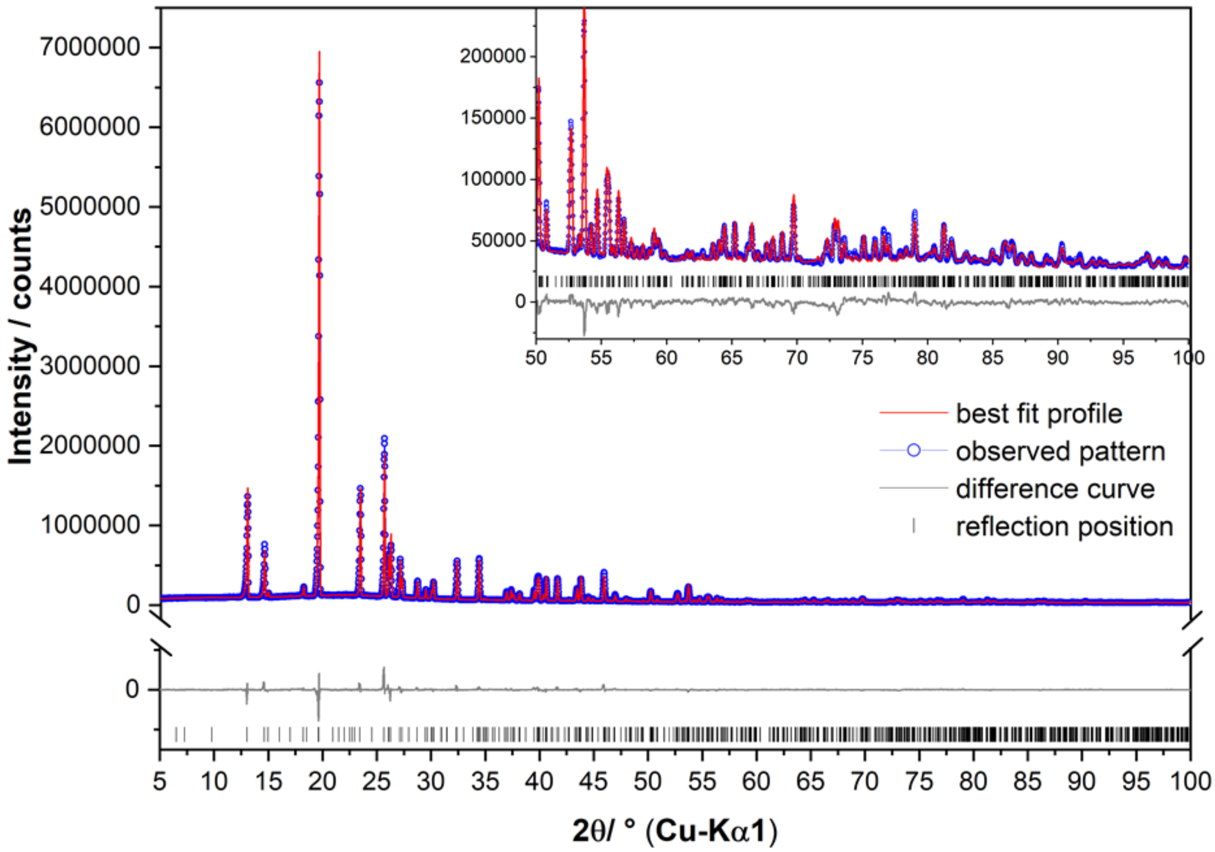

Figure S19. Rietveld plot of the model with *P*1 space group obtained from PDF refinement. The observed pattern (circles), the best Rietveld fit profile (line) and the difference curve between the observed and the calculated profiles (below) are shown. The high angle part starting at 50° in 2θ is enlarged for clarity.

Table S2. Crystallographic and Rietveld Refinement data of 1 using the models obtained from PDF refinements.

Compound	Model 1	Model 2	Model 3
**Crystal system**	Orthorhombic	Monoclinic	Triclinic
**Space group**	*Ama*2	*C*2	*P*1
**Wavelength / Å**	1.5406	1.5406	1.5406
***a* / Å**	13.5543(2)	13.5550(2)	13.5545(2)
***b* / Å**	5.20768(8)	5.20795(7)	5.20783(6)
***c* / Å**	12.1059(2)	12.1064(2)	12.1061(1)
**V / Å^3**	854.52(2)	854.64(2)	854.56(2)
**R_{wp} / % [a]**	6.35	6.08	5.53
**R_p / % [a]**	5.54	4.32	4.11
**R_{Bragg} / % [a]**	5.26	5.14	4.67
**Starting angle measured/ ° 2θ**	0	0	0
**Final angle measured / ° 2θ**	110	110	110
**Starting angle used / ° 2θ**	5	5	5
**Final angle used / ° 2θ**	100	100	100
**Step width / ° 2θ**	0.01	0.01	0.01

Table S3. Atomic coordinates of **1**.

Atom	Wyck.	Site symmetry	S.O.F.	*x/a*	*y/b*	*z/c*	B /Å²
C1	*4b*	m..	1	1/4	0.16077(4)	0.00302(15)	2.05(4)
C2	*8c*	1	1	0.34991(8)	0.31422(12)	0.0053(5)	2.05(4)
N1	*8c*	1	1	0.35158(9)	0.21088(4)	-0.0820(4)	2.05(4)
N2	*4b*	m..	1	1/4	0.36918(16)	-0.0695(5)	2.05(4)
C3	*4b*	m..	1	1/4	0.05301(0)	-0.0832(5)	2.05(4)
C4	*8c*	1	1	0.45271(8)	0.36798(12)	-0.0917(8)	2.05(4)
O1	*4b*	m..	1	1/4	0.0605(2)	-0.3597(4)	2.05(4)
O2	*8c*	1	1	0.4414(3)	0.3625(2)	-0.3672(8)	2.05(4)
H1	*4b*	m..	1	1/4	0.1581(12)	0.1911(2)	2.05(4)
H2	*8c*	1	1	0.3530(12)	0.3137(6)	0.1934(5)	2.05(4)
H3	*8c*	1	1	0.3521(14)	0.2125(7)	-0.2471(4)	2.05(4)
H4	*4b*	m..	1	1/4	0.3718(14)	-0.2345(5)	2.05(4)
H5	*8c*	1	1	0.31782(5)	0.01984(3)	-0.0165(6)	2.05(4)
H6	*8c*	1	1	0.51996(8)	0.33460(13)	-0.0331(10)	2.05(4)
H7	*8c*	1	1	0.45229(8)	0.43672(12)	-0.0333(10)	2.05(4)

Table S4. Selected atomic distances of **1**.

Atoms	Distance
C(1)-N(1)	1.4729(12) Å
C(1)-C(3)	1.528(1) Å
C(2)-N(1)	1.473(2) Å
C(2)-N(2)	1.473(2) Å
C(2)-C(4)	1.528(2) Å
C(3)-O(1)	1.443(3) Å
C(4)-O(2)	1.443(6) Å

4. XRPD/PDF co-refinement

In this work, the laboratory XRPD pattern and synchrotron PDF were analysed by refining parameters common for both datasets, such as lattice parameters, translations, rotations, bond lengths, torsions and thermal displacements. Before the co-refinement can be carried out, both datasets must be weighted in order to equally contribute to the refinement. This means finding a weighting factor for XRPD pattern and/or PDF where both methods can give together the best result. In the present work, we performed refinements with different weighting factors applied to the PDF dataset. Figure 19 shows the PDF and XRPD specific R_w values for each weighting during refinement with *C*2 space group. The best weighting factor for our data (value=10^5), which was used for XRPD/PDF co-refinement (black circle), corresponds to approximately $|I_{xrpd}|/(|I_{pdf}|\cdot 10)$, where I_{XRPD} is the integrated intensity of the XRPD pattern and I_{pdf} for the PDF plot)

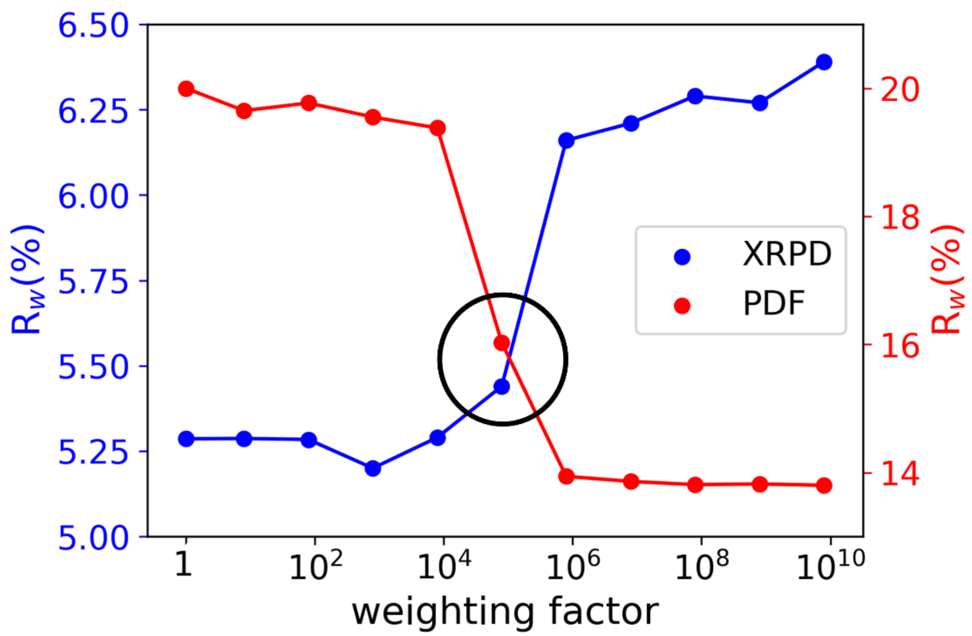

Figure S20. Weighting factor applied to PDF data and used for co-refinement versus agreement factor (R_w) obtained from XRPD (blue) and PDF (red) refinement with the model in *C*2 space group.

References

(1) Coelho, A. A.; Chater, P. A.; Kern, A. Fast synthesis and refinement of the atomic pair distribution function. *Journal of Applied Crystallography* **2015**, *48*, 869.
(2) Juhás, P.; Farrow, Christopher L.; Yang, X.; Knox, Kevin R.; Billinge, Simon J. L. Complex modeling: a strategy and software program for combining multiple information sources to solve ill posed structure and nanostructure inverse problems. *Acta Crystallographica Section A Foundations and Advances* **2015**, *71*, 562.
